# Inflammation and bone mineral density: A Mendelian randomization study

**DOI:** 10.1038/s41598-017-09080-w

**Published:** 2017-08-17

**Authors:** Jian V. Huang, C. Mary Schooling

**Affiliations:** 1 School of Public Health, Li Ka Shing Faculty of Medicine, The University of Hong Kong, Hong Kong SAR, People’s Republic of China; 2 City University of New York, Graduate School of Public Health and Health Policy, New York, USA

## Abstract

Osteoporosis is a common age-related disorder leading to an increase in osteoporotic fractures and resulting in significant suffering and disability. Inflammation may contribute to osteoporosis, as it does to many other chronic diseases. We examined whether inflammation is etiologically relevant to osteoporosis, assessed from bone mineral density (BMD), as a new potential target of intervention, or whether it is a symptom/biomarker of osteoporosis. We obtained genetic predictors of inflammatory markers from genome-wide association studies and applied them to a large genome wide association study of BMD. Using two-sample Mendelian randomization, we obtained unconfounded estimates of the effect of high-sensitivity C-reactive protein (hsCRP) on BMD at the forearm, femoral neck,﻿ an﻿d lumbar spine. After removing potentially pleiotropic single nucleotide polymorphisms (SNPs) possibly acting via obesity-related traits, hsCRP, based on 16 SNPs from genes including *CRP*, was not associated with BMD. A causal relation of hsCRP with lower BMD was not evident in this study.

## Introduction

Osteoporosis increases the risk of fracture. Bone mineral density (BMD), a biomarker of osteoporosis, is a risk factor for fracture which contributes to the global burden of disease from falls^1^. Between 1990 and 2010, global deaths and disability-adjusted life years attributable to low BMD increased, probably due to the aging global population^1^. The burden of osteoporosis (BMD 2.5 standard deviations (SDs) below the reference, i.e. the mean in healthy young adult women^2^) and osteopenia (BMD 1.0–2.5 SDs below the reference)^2^ among older adults is projected to rise in the coming decade, for example in the United States and Australia^3, 4^. Apart from the effects on population health, this escalating global burden of osteoporotic disorders places an increasing burden on healthcare providers^4–6^, because falls and fractures in older people can generate long hospital stays, extensive convalescence and permanent disability. Inflammation plays an important role in many major non-communicable diseases^7–9^, and is increasingly realized to be relevant to the bone remodeling^10^ that precedes osteoporosis^11^.

In a recent epidemiological study, older men (aged 65 years or above) with three or more inflammatory markers in the top quartile had a higher risk of hip or vertebral fracture, but the specific associations of each inflammatory marker with fracture at different skeletal sites varied^12^. Observational studies have reported associations of several major inflammatory markers, such as C-reactive protein (CRP), interleukin-6 (IL-6) and monocyte chemotactic protein-1 (MCP-1), with osteoporosis and/or osteopenia^13–15^, but findings have not always been consistent^16, 17^. Moreover, distinguishing between biomarkers and causal factors is difficult in observational studies, making it unclear whether inflammation is etiologically relevant to osteoporosis as a new potential target of intervention, is a symptom or is a correlate of other unmeasured causal factors. The controversy over current precautionary treatments for low BMD, such as calcium^18^, demonstrates the importance of thorough validation of targets of intervention, as well as the importance of the discovery of new targets.

In this situation assessing whether people with genetically higher levels of inflammatory markers have lower BMD, i.e., Mendelian randomization (MR), provides a way forward. Given genotypes are randomly assorted at conception, MR, or instrumental variable analysis with genetic variants, can mimic a randomized controlled trial and is becoming an important tool for assessing causal relations in observational studies^19, 20^. To the best of our knowledge, only one previous MR study has assessed the role of inflammation in BMD and fractures. It did not find a causal relation of higher high-sensitivity CRP (hsCRP) with higher fracture risk, suggesting hsCRP may be a marker of unknown confounders that affect fracture risk^21^. We used a two-sample MR study to estimate the unconfounded associations of several inflammatory markers with BMD. Specifically, we obtained genetic predictors of inflammatory markers from genome-wide association studies (GWAS) and applied them to a GWAS of BMD from a large international consortium. Inflammation and obesity have a complex interplay, which a bidirectional MR study clarified as adiposity likely causing higher CRP rather than CRP causing adiposity^22^. As such, we also checked whether the genetic predictors of inflammation also affected adiposity, to exclude potentially pleiotropic effects.

## Methods

### Genetic predictors of inflammatory markers

SNPs predicting inflammatory markers, including hsCRP, IL-6, erythrocyte sedimentation rate (ESR) and MCP-1, were obtained from SNPs of genome-wide significance (p < 5 × 10^−8^) from the largest available published GWAS. Correlations between these SNPs (linkage disequilibrium (LD)) were obtained from SNP Annotation and Proxy Search (SNAP) (http://www.broadinstitute.org/mpg/snap/ldsearchpw.php) using the 1000 genomes reference panel. Highly correlated SNPs (r^2^ ≥ 0.8) for each exposure were discarded to retain SNPs with the smallest p-values. A SNP highly correlated with the original SNP was used as proxy when the original SNP was not available in GEFOS. When the remaining SNPs for an exposure were correlated an LD matrix was built and included in the MR analysis to account for that correlation. MR studies assume that the SNPs (instrumental variables) are associated with the outcome only via the exposure^23^, so we performed sensitivity analysis excluding SNPs with potentially pleiotropic effects. Ensembl (http://www.ensembl.org) integrates genome annotation with available biological data, and provides the phenotypes associated with a given SNP. We manually searched Ensembl for the phenotypes associated with each SNP. SNPs with phenotypes that may affect BMD other than via the relevant exposure were considered as having potentially pleiotropic effects.

### Genetic predictors of BMD

Genetic associations with BMD were contributed by the GEnetic Factors for OSteoporosis (GEFOS) Consortium (http://www.gefos.org). The GEFOS-seq project investigated the associations of SNP with BMD in a general population of European descent (N = 32,965)^24^. Genetic associations with BMD for SNPs with a minor allele frequency ≥ 0.5% in an additive model adjusted for age, age-squared, sex, and weight^24^ were made publically available in 2015^25^. BMD was measured using Dual-energy X-ray Absorptiometry at three skeletal sites, the forearm (distal 1/3 of radius), lumbar spine (L1-4) and femoral neck^24^. BMD within each study was standardized to account for systematic differences between Dual-energy X-ray Absorptiometry machines^24^.

### Statistical Analysis

SNPs predicting the exposures (i.e. inflammatory markers) were used as instrumental variables in two-sample MR to obtain unconfounded estimates of the effects of hsCRP, IL-6, ESR, and MCP-1 on BMD (SD units) at the forearm, femoral neck, and lumbar spine. To obtain an overall estimate for each exposure, we combined SNP-specific Wald estimates using inverse variance weighting with the standard error calculated as the ratio of the standard error of SNP on outcome to the estimate of SNP on exposure. We used fixed effects when only three or fewer SNPs were available, and used random effects when four or more SNPs were available. Where SNPs for a particular exposure were correlated, we used generalized weighted linear regression and a matrix of their correlations. In order to assess the robustness of these findings, we used a weighted median (WM) estimate, which is consistent when up to 50% of the SNPs are invalid instrumental variables^26^. We also used MR-Egger to test for potentially pleiotropic effects as it may generate correct estimates even when all SNPs are invalid instruments as long as the assumption of instrument strength independent of direct effect (InSIDE) is satisfied. Average directional pleiotropy across genetic variants was assessed from the p-value of the intercept term from MR-Egger^26^. Analysis using MR-Egger has a lower false positive rate but a higher false negative rate than IVW^27^. We also similarly checked the associations of the SNPs predicting inflammation with obesity-related traits from the largest available GWAS among people of European descent in the Genetic Investigation of ANthropometric Traits (GIANT) consortium databases^28–30^, and Early Growth Genetics (EGG) consortium database^31^. SNPs with potentially pleiotropic effects were excluded in an additional analysis. We used a p-value of 0.0125 as significance threshold, because we performed MR analysis for four inflammatory markers, i.e. hsCRP, IL-6, ESR, and MCP-1.

All statistical analyses were conducted using R version 3.3.0 (R Foundation for Statistical Computing, Vienna, Austria) and MR analyses were performed using the MendelianRandomization package. This study only used publicly available summary data and hence no ethical approval from an Institutional Review Board was required.

## Results

The largest available GWAS for hsCRP identified 20 SNPs (from genes including *CRP*, *IL-6R*, and *LEPR*) predicting natural log-transformed CRP from two studies of 194,418 (mean age 59 years)^32^ and 66,185 (mean age 55 years)^33^ people of European descent^34^. Among these 20 SNPs, 6 proxy SNPs were used, i.e., rs3116656 (Intergenic variant) as a proxy SNP for rs1130864 (*CRP*), rs11065385 (*HNF1A*) for rs1183910 (*HNF1A*), rs780094 (*GCKR*) for rs1260326 (*GCKR*), rs77013776 (intergenic variant) for rs1800947 (*CRP*), rs61812598 (*IL6R*) for rs4129267 (*IL6R*), and rs993394 (*GPRC6A*) for rs6901250 (*GPRC6A*).

A GWAS by Naitza *et al*. provides genetic predictors of IL-6, ESR, and MCP-1 (in SD units) based on 4292, 3596, and 4295 people respectively aged 14–102 years from the Lanusei Valley of Sardinia, Italy^35, 36^. Four SNPs from *ABO* were used to predict IL-6 (3 SNPs in the analysis of each skeletal site). Two proxy SNPs were used, i.e. rs657152 (*ABO*) as a proxy for rs643434 (*ABO*) and rs544873 as a proxy for rs687289 (*ABO*). However, highly pleiotropic effects of *ABO* cast doubt on whether these SNPs solely affect BMD via IL-6. We repeated the analysis for IL-6 using SNPs from two other sources. Three SNPs in *IL6R*, predicting natural log-transformed IL-6, were obtained from a previous MR study by the IL-6R MR Analysis Consortium in 4489 Europeans^37^. rs61812598 (*IL6R*) was used as a proxy for rs7529229 in the analysis of BMD at the femoral neck and lumbar spine. Another three SNPs predicting IL-6 (from *KPNB1*, *SERPINE2*, *TC2N*) were obtained from a GWAS by Ahola-Olli *et al*. in 1664 Finns^38^. Four SNPs from *CR1* were used to predict ESR, of which 3 were included in the analysis of BMD at the femoral neck and lumbar spine respectively. Twenty-three SNPs were used to predict MCP-1 (from *ACKR1*, *AIM2, CADM3*, *DUSP23*, *FCER1A*, *OR10J1*, *OR10J2P*, *OR10J7P*, *OR10J9P*, and intergenic variant), among which 22 were included in the analysis of BMD at the femoral neck and lumbar spine respectively. rs79433881 (*OR10J1*) was used as a proxy for rs11265186 (*OR10J9P*) in the analysis of BMD at the femoral neck, and rs4290055 was used as a proxy for rs11265186 (*OR10J9P*) in the analysis of BMD at the lumbar spine.

We matched the chosen SNPs with GWAS of obesity-related traits and found none of the SNPs achieved genome-wide significance in GIANT or EGG (Table 1 in the Supplementary material). Nevertheless, some of these SNPs (rs1260326 (*GCKR*), rs13233571 (*BCL7B*), rs2847281 (*PTPN2*), and rs4129267 (*IL6R*) predicting hsCRP and rs630014 (*ABO*), rs651007 (*ABO*), and rs687289 (*ABO*) predicting IL-6) were associated with obesity-related traits (Table 2 in the Supplementary material). None of the individual SNPs predicting MCP-1 were associated with obesity-related traits, but the IVW estimate showed potential associations of MCP-1 with higher BMI in men and higher risk of obesity class 3 (Table 3 in the Supplementary material). rs12075 (*DARC*/*CADM3*) that predicted MCP-1 also has potential pleiotropic effects on obesity-related traits based on a comprehensive search of Ensembl.

**Figure 1:**
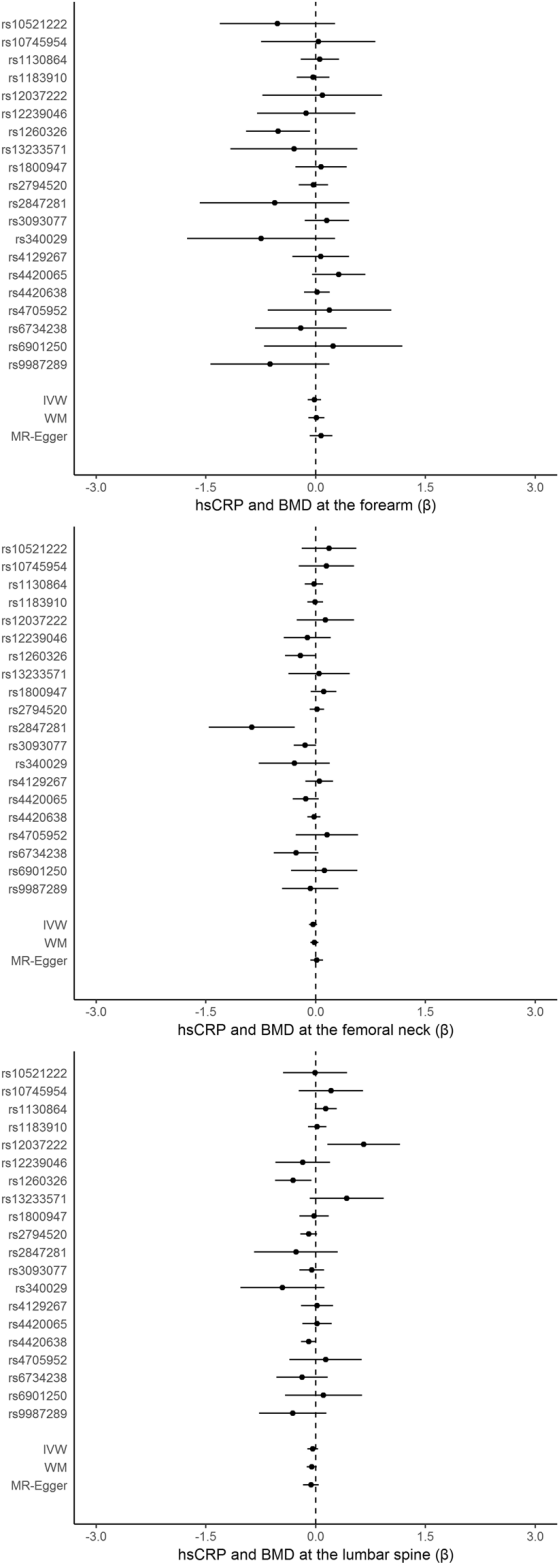
SNP-specific associations of hsCRP (Prins *et al*.) with bone mineral density (BMD) at each skeletal site.

Funnel plots for hsCRP, IL-6, ESR, and MCP-1 show some asymmetry (Figures 1 to 4 in the Supplementary material). Figures 5 to 8 in the Supplementary material show the scatter plots for hsCRP, IL-6, ESR, and MCP-1. We performed analyses including all available SNPs and sensitivity analyses excluding SNPs with potentially pleiotropic effects such as SNPs in the pleiotropic genes *GCKR* and *ABO*. Table 1 shows the estimates of the effects on BMD. hsCRP was not associated with BMD in any model. Figure 1 shows the SNP-specific associations and MR estimates for hsCRP. Considering the limitations of the SNPs predicting IL-6, ESR, and MCP-1, estimates for these exposure are only presented in Table 4 and Figures 9 to 11 in the Supplementary material. MCP-1 was associated with lower BMD at the forearm, but with higher BMD at the femoral neck and lumbar spine. However, the association of MCP-1 with BMD at the forearm was no longer evident after removing rs12075 (*DARC*/*CADM3*) in a sensitivity analysis (Table 4 in the Supplementary material). Intercepts from MR-Egger showed little directional pleiotropy, but the direction of effect estimates was not always consistent for both IVW and WM.

**Table 1:** Estimates of the effects of hsCRP on bone mineral density (in standard deviations) at forearm, femoral neck, and lumbar spine provided by GEFOS.

Inflammatory marker	GWAS^a^	Skeletal site (GEFOS 2015)^b^	All SNPs						Excluding potentially pleiotropic SNPs^f^					
			No. of SNPs^c^	Method^d^	β	P-value^e^	MR-Egger		No. of SNPs^b^	Method	β	P-value^e^	MR-Egger	
							Intercept	P-value					Intercept	P-value
hsCRP	Prins *et al*.	Forearm	20	IVW	−0.018	0.692			16	IVW	0.009	0.857		
			20	WM	0.011	0.838			16	WM	0.012	0.828		
			20	MR-Egger	0.076	0.337	−0.011	0.144	16	MR-Egger	0.054	0.506	−0.006	0.487
		Femoral neck	20	IVW	−0.035	0.215			16	IVW	−0.025	0.298		
			20	WM	−0.016	0.580			16	WM	−0.016	0.574		
			20	MR-Egger	0.015	0.729	−0.006	0.158	16	MR-Egger	−0.014	0.726	−0.001	0.749
		Lumbar spine	20	IVW	−0.038	0.298			16	IVW	−0.032	0.402		
			20	WM	−0.054	0.119			16	WM	−0.056	0.121		
			20	MR-Egger	−0.064	0.237	0.003	0.560	16	MR-Egger	−0.074	0.184	0.005	0.355

^a^In the MR study of Prins *et al*., hsCRP was analysed on a natural log scale in mg/L.

^b^Bone mineral density was measured as the standard deviation (SD) from the healthy young adult reference.

^c^Number of SNPs included in the analysis of one markers at different skeletal sites may differ, because particular SNPs may not be available in GEFOS of specific skeletal site.

^d^Inverse-variance weighted (IVW), weighted median (WM), and MR-Egger were performed for testing the robustness of the association; WM and MR-Egger are only feasible with more than two SNPs.

^e^Associations with a p-value smaller than 0.0125 were in bold.

^f^SNPs with potentially pleiotropic effects related to obesity were excluded from the sensitivity analysis, including rs1260326 (*GCKR*), rs13233571 (*BCL7B*), rs2847281 (*PTPN2*), and rs4129267 (*IL6R*) for hsCRP.

## Discussion

For the first time, we assessed the associations of four major inflammatory markers (i.e. hsCRP, IL-6, ESR, and MCP-1) with BMD using two-sample MR to provides unconfounded estimates because genotypes are randomly assorted at conception and usually unaffected by the confounders that typically bedevil epidemiological studies, such as socio-economic position and health status. Use of two different samples for the exposures and outcomes also minimizes susceptibility to confounding because of the different underlying data structures in each sample^39^. We only used SNPs strongly associated with inflammatory markers from the largest available GWAS to reduce the risk of false positives, and we used LD matrices to account for any correlation between SNPs predicting each exposure. We also checked for potentially pleiotropic effects and removed them in sensitivity analyses. We did not observe associations of higher hsCRP with lower BMD.

Although we checked the assumptions of MR rigorously, some limitations exist. First, genetic predictors of IL-6, ESR, and MCP-1 were based on an isolated population in the Lanusei Valley of Sardinia, Italy^36^. These results may be susceptible to population stratification and might not be applicable to other populations. In addition, no more than 4 SNPs were available for IL-6 and ESR, meaning the results for these exposures should be interpreted with caution. However, we repeated the analyses for IL-6 using SNPs from IL-6R MR Analysis Consortium and the GWAS by Ahola-Olli *et al*., and found similar results. Second, the specific effect of SNPs on a phenotype may be compensated for during development, i.e. canalisation, meaning that the effect of an intervention might not mirror the genetic association^20^, however whether such canalisation exists is unknown. Third, estimates may be sensitive to the choice of MR estimator. To test robustness, we also used WM and MR-Egger methods. The estimates were generally similar, but MR-Egger estimates had much wider confidence intervals, and WM with 3 or 4 SNPs may not return meaningful results. Fourth, statistical power of MR studies needs particular consideration, given that genetic instruments may only explain a small amount of variation of the phenotype^40^. We were unable to calculate the power for this study because the GWAS of Naitza *et al*. and of Prins *et al*. did not report the explanation power of SNPs on inflammatory markers. However, one advantage of two-sample MR is increased statistical power^41^. We also estimated the F-statistics based on the effect allele frequency, estimated SNP effect on exposure, and sample size^42^. F-statistics range from 32 to 4419, except for the three SNPs from Ahola-Olli *et al*., which had F-statistics ranging from 3 to 8 (Table 5 in the Supplementary material). An F-statistic higher than the conventional threshold of 10 is usually taken as implying little weak instrument bias. Fifth, associations of higher genetically predicted MCP-1 with lower BMD were only observed at the femoral neck and lumbar spine. Given that BMD is likely to be correlated at different skeletal sites, this may suggest chance findings. Sixth, local and systemic inflammation may impact bone growth via different pathways^43^, which we could not distinguish. Seventh, GEFOS is based on the general population, but osteoporosis is more prevalent among the elderly. However, bone loss may start in middle age and increase the risk of osteoporosis and fracture in later life. Lastly, while the prevalence of osteoporosis is higher in women, sex-specific analysis was not possible in this study, although causal relations are usually consistent.

Previous observational studies have reported inconsistent associations of inflammatory markers with BMD^13, 16, 17, 44^. Inflammation may affect BMD via the bone remodeling cycle regulated by osteoblasts and osteoclasts^45^ affecting osteocalcin^46^. Previous studies have observed inverse associations of hsCRP with serum osteocalcin^47, 48^, which may play a role in osteoclastogenesis^49^. However, an *in vitro* study suggested that CRP inhibits both osteoblast and osteoclast differentiation^50^. Hence, the impact of these inflammatory markers on BMD is unclear. SNPs predicting hsCRP with potentially pleiotropic effects via obesity-related traits were identified in our study, which may imply that the previously reported associations of inflammation with BMD are not causal. Also, higher follicle-stimulating hormone is associated with both bone loss^51^ and higher levels of inflammatory markers including CRP and MCP-1^52^, implying a possible confounder of the association of inflammation with bone loss.

A causal relationship of hsCRP with lower BMD was not evident in this study. A replication in the UK Biobank would help clarify whether these null findings are due to lack of power or indicate no effect and that other means of preventing osteoporosis should be sought.

## Electronic supplementary material

Supplementary material

## References

[1] Sanchez-Riera L (2014). The global burden attributable to low bone mineral density. Annals of the rheumatic diseases.

[2] Kanis JA (2008). A reference standard for the description of osteoporosis. Bone.

[3] Wright NC (2014). The recent prevalence of osteoporosis and low bone mass in the United States based on bone mineral density at the femoral neck or lumbar spine. Journal of bone and mineral research: the official journal of the American Society for Bone and Mineral Research.

[4] Watts, J. J., Abimanyi-Ochom, J. & Sanders, K. M. Osteoporosis costing all Australians A new burden of disease analysis - 2012 to 2022. (Osteoporosis Australia, Australia, 2012).

[5] Hopkins, R. B. *et al*. The current economic burden of illness of osteoporosis in Canada. *Osteoporosis international: a journal established as result of cooperation between the European Foundation for Osteoporosis and the National Osteoporosis Foundation of the USA*, doi:10.1007/s00198-016-3631-6 (2016). 10.1007/s00198-016-3631-6 PMC5104559 27166680

[6] Burge R (2007). Incidence and economic burden of osteoporosis-related fractures in the United States, 2005–2025. Journal of bone and mineral research: the official journal of the American Society for Bone and Mineral Research.

[7] Heppner FL, Ransohoff RM, Becher B (2015). Immune attack: the role of inflammation in Alzheimer disease. Nature reviews. Neuroscience.

[8] Eizirik DL, Colli ML, Ortis F (2009). The role of inflammation in insulitis and beta-cell loss in type 1 diabetes. Nature reviews. Endocrinology.

[9] Medzhitov R (2008). Origin and physiological roles of inflammation. Nature.

[10] Loi F (2016). Inflammation, fracture and bone repair. Bone.

[11] Tilg H, Moschen AR, Kaser A, Pines A, Dotan I (2008). Gut, inflammation and osteoporosis: basic and clinical concepts. Gut.

[12] Cauley JA (2016). Inflammatory Markers and the Risk of Hip and Vertebral Fractures in Men: the Osteoporotic Fractures in Men (MrOS). Journal of bone and mineral research: the official journal of the American Society for Bone and Mineral Research.

[13] Sponholtz TR (2014). Association Between Inflammatory Biomarkers and Bone Mineral Density in a Community-Based Cohort of Men and Women. Arthrit Care Res.

[14] Scheidt-Nave C (2001). Serum interleukin 6 is a major predictor of bone loss in women specific to the first decade past menopause. J Clin Endocr Metab.

[15] Eraltan H (2012). MCP-1 and CCR2 gene variants and the risk for osteoporosis and osteopenia. Genetic testing and molecular biomarkers.

[16] Berglundh S, Malmgren L, Luthman H, McGuigan F, Akesson K (2015). C-reactive protein, bone loss, fracture, and mortality in elderly women: a longitudinal study in the OPRA cohort. Osteoporosis international: a journal established as result of cooperation between the European Foundation for Osteoporosis and the National Osteoporosis Foundation of the USA.

[17] Kania DM (1995). Elevated plasma levels of interleukin-6 in postmenopausal women do not correlate with bone density. Journal of the American Geriatrics Society.

[18] Tai V, Leung W, Grey A, Reid IR, Bolland MJ (2015). Calcium intake and bone mineral density: systematic review and meta-analysis. Bmj.

[19] Burgess S (2015). Using published data in Mendelian randomization: a blueprint for efficient identification of causal risk factors. European journal of epidemiology.

[20] Haycock PC (2016). Best (but oft-forgotten) practices: the design, analysis, and interpretation of Mendelian randomization studies. The American journal of clinical nutrition.

[21] Oei L (2014). Dissecting the relationship between high-sensitivity serum C-reactive protein and increased fracture risk: the Rotterdam Study. Osteoporosis Int.

[22] Welsh P (2010). Unraveling the directional link between adiposity and inflammation: a bidirectional Mendelian randomization approach. The Journal of clinical endocrinology and metabolism.

[23] Lawlor DA, Harbord RM, Sterne JA, Timpson N, Davey Smith G (2008). Mendelian randomization: using genes as instruments for making causal inferences in epidemiology. Stat Med.

[24] Zheng HF (2015). Whole-genome sequencing identifies EN1 as a determinant of bone density and fracture. Nature.

[25] Genetic Factors for Osteoporosis (GEFOS) Consortium. *Data Release 2015*, http://www.gefos.org/?q=content/data-release-2015 (2016).

[26] Bowden J, Smith GD, Haycock PC, Burgess S (2016). Consistent Estimation in Mendelian Randomization with Some Invalid Instruments Using a Weighted Median Estimator. Genet Epidemiol.

[27] Burgess, S., Bowden, J., Fall, T., Ingelsson, E. & Thompson, S. G. Sensitivity analyses for robust causal inference from Mendelian randomization analyses with multiple genetic variants. *Epidemiology* (2016). 10.1097/EDE.0000000000000559 PMC5133381 27749700

[28] Berndt SI (2013). Genome-wide meta-analysis identifies 11 new loci for anthropometric traits and provides insights into genetic architecture. Nat. Genet..

[29] Randall JC (2013). Sex-stratified genome-wide association studies including 270,000 individuals show sexual dimorphism in genetic loci for anthropometric traits. PLoS genetics.

[30] Locke AE (2015). Genetic studies of body mass index yield new insights for obesity biology. Nature.

[31] Bradfield JP (2012). A genome-wide association meta-analysis identifies new childhood obesity loci. Nat. Genet..

[32] Wensley, F. *et al*. Association between C reactive protein and coronary heart disease: mendelian randomisation analysis based on individual participant data. *Brit Med J* **342**, doi:10.1136/bmj.d548 (2011). 10.1136/bmj.d548 PMC3039696 21325005

[33] Dehghan A (2011). Meta-analysis of genome-wide association studies in >80 000 subjects identifies multiple loci for C-reactive protein levels. Circulation.

[34] Prins BP (2016). Investigating the Causal Relationship of C-Reactive Protein with 32 Complex Somatic and Psychiatric Outcomes: A Large-Scale Cross-Consortium Mendelian Randomization Study. PLoS medicine.

[35] Pilia G (2006). Heritability of cardiovascular and personality traits in 6,148 Sardinians. PLoS genetics.

[36] Naitza S (2012). A genome-wide association scan on the levels of markers of inflammation in Sardinians reveals associations that underpin its complex regulation. PLoS genetics.

[37] Consortium I-RMRA (2012). The interleukin-6 receptor as a target for prevention of coronary heart disease: a mendelian randomisation analysis. Lancet.

[38] Ahola-Olli AV (2017). Genome-wide Association Study Identifies 27 Loci Influencing Concentrations of Circulating Cytokines and Growth Factors. Am. J. Hum. Genet..

[39] Inoue A, Solon G (2010). Two-Sample Instrumental Variables Estimators. Rev Econ Stat.

[40] von Hinke Kessler Scholder S, Smith GD, Lawlor DA, Propper C, Windmeijer F (2011). Mendelian randomization: the use of genes in instrumental variable analyses. Health Econ..

[41] Lawlor DA (2016). Commentary: Two-sample Mendelian randomization: opportunities and challenges. International journal of epidemiology.

[42] Del Greco MF (2017). Serum iron level and kidney function: a Mendelian randomization study. Nephrol. Dial. Transplant..

[43] Skerry, T. M. The effects of the inflammatory response on bone growth. *Eur. J. Clin. Nutr*. **48** Suppl 1, S190–197; discussion S198 (1994). 8005086

[44] Lim HS, Park YH, Kim SK (2016). Relationship between Serum Inflammatory Marker and Bone Mineral Density in Healthy Adults. Journal of bone metabolism.

[45] Hardy R, Cooper MS (2009). Bone loss in inflammatory disorders. J Endocrinol.

[46] Cantatore FP (2004). Osteocalcin synthesis by human osteoblasts from normal and osteoarthritic bone after vitamin D3 stimulation. Clinical rheumatology.

[47] Sarkar PD, Choudhury AB (2013). Relationships between serum osteocalcin levels versus blood glucose, insulin resistance and markers of systemic inflammation in central Indian type 2 diabetic patients. Eur Rev Med Pharmaco.

[48] Chen L (2013). Osteocalcin, glucose metabolism, lipid profile and chronic low-grade inflammation in middle-aged and elderly Chinese. Diabetic medicine: a journal of the British Diabetic Association.

[49] Sabokbar A, Mahoney DJ, Hemingway F, Athanasou NA (2016). Non-Canonical (RANKL-Independent) Pathways of Osteoclast Differentiation and Their Role in Musculoskeletal Diseases. Clinical reviews in allergy & immunology.

[50] Cho IJ (2016). Effects of C-reactive protein on bone cells. Life sciences.

[51] Liu P (2017). Blocking FSH induces thermogenic adipose tissue and reduces body fat. Nature.

[52] Kass AS, Lea TE, Torjesen PA, Gulseth HC, Forre OT (2010). The association of luteinizing hormone and follicle-stimulating hormone with cytokines and markers of disease activity in rheumatoid arthritis: a case-control study. Scand. J. Rheumatol..

