# Supplementary material for: Inflammation and bone mineral density: A Mendelian randomization study
Source: Sci Rep. 2017 Aug 17;7:8666. doi: 10.1038/s41598-017-09080-w (PMC5561220; doi:10.1038/s41598-017-09080-w)
Supplement: Supplementary file 1 — Supplementary material [file 41598_2017_9080_MOESM1_ESM.doc]

**Supplementary material**

**Inflammation and bone mineral density: A Mendelian randomization study**

**Jian V Huang, C Mary Schooling**

Table 1 Associations of single nucleotide polymorphisms (SNPs) predicting inflammatory markers with obesity-related outcomes from the Genetic Investigation of ANthropometric Traits (GIANT) consortium databases[^1-3^](#_ENREF_1) and Early Growth Genetics (EGG) consortium database.[^4^](#_ENREF_4)

| **Inflammatory marker** | **Source** | **SNP** | **P-value** | | | | | | | | | |
| --- | --- | --- | --- | --- | --- | --- | --- | --- | --- | --- | --- | --- |
|  |  |  | **Obesity, overweight**[**^1^**](#_ENREF_1) | **Obesity, class1**[**^1^**](#_ENREF_1) | **Obesity, class2**[**^1^**](#_ENREF_1) | **Obesity, class3**[**^1^**](#_ENREF_1) | **Childhood obesity**[**^4^**](#_ENREF_4) | **Weight, men**[**^2^**](#_ENREF_2) | **Weight, women**[**^2^**](#_ENREF_2) | **BMI, men**[**^3^**](#_ENREF_3) | **BMI, women**[**^3^**](#_ENREF_3) | **Extreme BMI**[**^1^**](#_ENREF_1) |
| hsCRP | Prins *et al.* | rs10521222 | 0.130 | 1.000 | 0.240 | NA | 0.245 | 0.230 | 0.710 | 0.624 | 0.658 | NA |
|  |  | rs10745954 | 0.150 | 0.100 | 0.100 | 0.092 | 0.850 | 0.056 | 0.530 | 0.017 | 0.610 | 0.250 |
|  |  | rs1130864 | 0.580 | 0.760 | 0.800 | 0.930 | 0.481 | 0.840 | 0.074 | 0.812 | 0.203 | 0.930 |
|  |  | rs1183910 | 0.780 | 0.930 | 0.760 | 0.770 | 0.926 | 0.750 | 0.790 | 0.818 | 0.620 | 0.940 |
|  |  | rs12037222 | 0.520 | 0.040 | 0.170 | 0.820 | 0.707 | 0.120 | 0.080 | 0.015 | 0.672 | 0.380 |
|  |  | rs12239046 | 0.590 | 0.880 | 0.800 | 0.870 | 0.768 | 0.840 | 0.930 | 0.795 | 0.484 | 0.880 |
|  |  | rs1260326 | 0.120 | 0.085 | 0.022 | 0.098 | 0.097 | 0.000 | 0.290 | 0.000 | 0.037 | 0.018 |
|  |  | rs13233571 | 0.890 | 0.860 | 0.004 | 0.190 | 0.386 | 0.940 | 0.530 | 0.215 | 0.126 | 0.130 |
|  |  | rs1800947 | 0.480 | 0.520 | 0.560 | 0.390 | 0.668 | 0.400 | 0.980 | 0.239 | 0.414 | NA |
|  |  | rs2794520 | 0.081 | 0.310 | 0.740 | 0.110 | 0.366 | 0.850 | 0.460 | 0.293 | 0.057 | 0.840 |
|  |  | rs2847281 | 0.002 | 0.009 | 0.012 | 0.059 | 0.434 | 0.720 | 0.110 | 0.011 | 0.009 | 0.086 |
|  |  | rs3093077 | 0.620 | 0.820 | 0.490 | 0.520 | 0.607 | 0.930 | 0.460 | 0.751 | 0.854 | NA |
|  |  | rs340029 | 0.050 | 0.068 | 0.078 | 0.400 | 0.596 | 0.440 | 0.011 | 0.073 | 0.005 | 0.005 |
|  |  | rs4129267 | 0.720 | 0.940 | 0.760 | 0.920 | 0.387 | 0.200 | 0.960 | 0.001 | 0.854 | 0.520 |
|  |  | rs4420065 | 0.730 | 0.460 | 0.730 | 0.100 | 0.429 | 0.630 | 0.240 | 0.477 | 0.403 | 0.440 |
|  |  | rs4420638 | 0.012 | 0.013 | 0.990 | 0.240 | 0.444 | 0.880 | 0.390 | 0.069 | 0.000 | 0.330 |
|  |  | rs4705952 | 0.890 | 0.790 | 0.260 | 0.480 | 0.856 | 0.780 | 0.920 | 0.223 | 0.816 | 0.500 |
|  |  | rs6734238 | 0.250 | 0.170 | 0.110 | 0.540 | 0.412 | 0.300 | 0.061 | 0.984 | 0.035 | 0.011 |
|  |  | rs6901250 | 0.690 | 0.230 | 0.370 | 0.510 | 0.175 | 0.900 | 0.920 | 0.195 | 0.217 | 0.310 |
|  |  | rs9987289 | 0.600 | 0.057 | 0.310 | 0.940 | 0.132 | 0.670 | 0.690 | 0.051 | 0.355 | 0.510 |
| IL-6 | Naitza *et al.* | rs579459 | 0.190 | 0.840 | 0.410 | 0.860 | 0.197 | 0.380 | 0.660 | 0.132 | 0.316 | 0.830 |
|  |  | rs630014 | 0.230 | 0.900 | 0.960 | 0.910 | 0.425 | 0.004 | 0.900 | 0.076 | 0.290 | 0.490 |
|  |  | rs643434 | 0.280 | 0.990 | 0.960 | 0.110 | 0.794 | 0.066 | 0.770 | NA | NA | 0.390 |
|  |  | rs651007 | 0.290 | 0.970 | 0.330 | 0.820 | 0.211 | 0.440 | 0.550 | 0.317 | 0.718 | 0.740 |
|  |  | rs687289 | 0.460 | 0.800 | 0.930 | 0.110 | 0.523 | 0.031 | 0.850 | NA | NA | 0.280 |
|  | IL6RMR | rs4845371 | 0.830 | 0.820 | 0.920 | 0.750 | 0.657 | 0.650 | 0.580 | 0.570 | 0.625 | 0.780 |
|  |  | rs7529229 | 0.100 | 0.550 | NA | NA | 0.447 | 0.400 | 0.550 | 0.801 | 0.242 | NA |
| ESR | Naitza *et al.* | rs11117956 | 0.016 | 0.011 | 0.230 | 0.650 | 0.993 | 0.640 | 0.490 | 0.781 | 0.228 | 0.910 |
|  |  | rs12034598 | 0.026 | 0.011 | 0.420 | 0.570 | 0.915 | 0.690 | 0.600 | 0.639 | 0.325 | 0.880 |
|  |  | rs3886100 | 0.350 | 0.320 | 0.220 | 0.580 | 0.736 | 0.930 | 0.630 | 0.597 | 0.631 | 0.390 |
|  |  | rs7519119 | 0.290 | 0.250 | 0.240 | 0.540 | 0.758 | 0.870 | 1.000 | 0.715 | 0.438 | 0.400 |
| MCP-1 | Naitza *et al.* | rs10489849 | 0.990 | 0.670 | 0.500 | 0.370 | 0.099 | 0.050 | 0.620 | 0.214 | 0.823 | 0.260 |
|  |  | rs11265142 | 0.400 | 0.100 | 0.950 | 0.200 | 0.337 | 0.350 | 0.440 | 0.621 | 0.092 | 0.220 |
|  |  | rs11265177 | 0.610 | 0.220 | 0.760 | 0.780 | 0.638 | 0.670 | 0.330 | 0.247 | 0.635 | 0.820 |
|  |  | rs11265186 | 0.670 | 0.640 | 0.460 | 0.490 | 0.271 | 0.290 | 0.081 | 0.632 | 0.611 | 0.620 |
|  |  | rs12047230 | 0.900 | 0.840 | 0.250 | 0.410 | 0.298 | 0.490 | 0.570 | 0.784 | 0.278 | 0.084 |
|  |  | rs12075 | 0.390 | 0.290 | 0.150 | 0.300 | 0.121 | 0.350 | 0.830 | 0.970 | 0.791 | 0.990 |
|  |  | rs12087465 | 0.420 | 0.500 | 0.960 | 0.850 | 0.847 | 0.900 | 0.760 | 0.991 | 0.374 | 0.160 |
|  |  | rs1446954 | 0.620 | 0.240 | 0.820 | 0.320 | 0.941 | 0.950 | 0.440 | 0.975 | 0.790 | 0.270 |
|  |  | rs1474747 | 0.610 | 0.130 | 0.510 | 0.880 | 0.289 | 0.110 | 0.740 | 0.257 | 0.185 | 0.880 |
|  |  | rs1584252 | 0.640 | 0.740 | 0.250 | 0.450 | 0.221 | 0.570 | 0.910 | 0.744 | 0.338 | 0.037 |
|  |  | rs16841987 | 0.930 | 0.730 | 0.900 | 0.700 | 0.057 | 0.110 | 0.520 | 0.174 | 0.366 | 0.230 |
|  |  | rs17666424 | 0.990 | 0.950 | 0.810 | 0.630 | 0.184 | 0.048 | 0.550 | 0.079 | 0.350 | 0.180 |
|  |  | rs2427825 | 0.590 | 0.360 | 0.760 | 0.220 | 0.305 | 0.610 | 0.990 | 0.470 | 0.636 | 0.770 |
|  |  | rs2494261 | 0.730 | 0.450 | 0.800 | 0.310 | 0.346 | 0.970 | 0.650 | 0.933 | 0.985 | 0.880 |
|  |  | rs2592881 | 0.250 | 0.150 | 0.430 | 0.860 | 0.934 | 0.330 | 0.910 | 0.498 | 0.278 | 0.240 |
|  |  | rs3026946 | 0.880 | 0.730 | 0.320 | 0.860 | 0.834 | 0.950 | 0.950 | 0.685 | 0.644 | 0.630 |
|  |  | rs3026968 | 0.860 | 0.820 | 0.770 | 0.790 | 0.708 | 0.660 | 0.450 | 0.505 | 0.902 | 0.850 |
|  |  | rs3027012 | 0.290 | 0.930 | 0.051 | 0.930 | 0.226 | 0.160 | 0.840 | 0.589 | 0.850 | 0.290 |
|  |  | rs3027031 | 0.130 | 0.220 | 0.098 | 0.950 | 0.939 | 0.032 | 0.055 | 0.256 | 0.033 | 0.870 |
|  |  | rs3806185 | 0.350 | 0.190 | 0.420 | 0.820 | 0.791 | 0.230 | 0.920 | 0.385 | 0.712 | 0.870 |
|  |  | rs4656237 | 0.690 | 0.590 | 0.530 | 0.510 | 0.229 | 0.400 | 0.085 | 0.814 | 0.650 | 0.550 |
|  |  | rs6660102 | 0.990 | 0.990 | 0.750 | 0.780 | 0.085 | 0.076 | 0.790 | 0.195 | 0.494 | 0.120 |

**BMI**: body mass index;

**Obesity, overweight**: BMI ≥25 (case) vs. BMI <25 (control);

**Obesity, class1**: BMI ≥30 (case) vs. BMI <25 (control);

**Obesity, class2**: BMI ≥35 (case) vs. BMI <25 (control);

**Obesity class 3**: BMI ≥40 (case) vs. BMI <25 (control);

**Childhood obesity**: ≥95th percentile of BMI (case) and <50th percentile of BMI (control);

**Extreme BMI**: upper 5th percentile (case) vs. lower 5th percentile (control).

**Table 1 (Cont.)** Associations of single nucleotide polymorphisms (SNPs) predicting inflammatory markers with obesity-related outcomes from the Genetic Investigation of ANthropometric Traits (GIANT) consortium databases[^1-3^](#_ENREF_1) and Early Growth Genetics (EGG) consortium database.[^4^](#_ENREF_4)

| **Inflammatory marker** | **Source** | **SNP** | **P-value** | | | | | | |
| --- | --- | --- | --- | --- | --- | --- | --- | --- | --- |
|  |  |  | **WHRadjBMI, men**[**^2^**](#_ENREF_2) | **WHRadjBMI, women**[**^2^**](#_ENREF_2) | **Extreme WHRadjBMI**[**^1^**](#_ENREF_1) | **HIPadjBMI, men**[**^2^**](#_ENREF_2) | **HIPadjBMI, women**[**^2^**](#_ENREF_2) | **WCadjBMI, men**[**^2^**](#_ENREF_2) | **WCadjBMI, women**[**^2^**](#_ENREF_2) |
| hsCRP | Prins *et al.* | rs10521222 | 0.270 | 0.370 | NA | 0.950 | 0.850 | 0.200 | 0.210 |
|  |  | rs10745954 | 0.710 | 0.520 | 0.760 | 0.570 | 0.540 | 0.830 | 0.530 |
|  |  | rs1130864 | 0.150 | 0.540 | 0.880 | 0.730 | 0.470 | 0.480 | 0.920 |
|  |  | rs1183910 | 0.890 | 0.760 | 0.600 | 0.520 | 0.200 | 0.260 | 0.240 |
|  |  | rs12037222 | 0.350 | 0.062 | 0.210 | 0.069 | 0.480 | 0.360 | 0.100 |
|  |  | rs12239046 | 0.510 | 0.590 | 0.580 | 0.052 | 0.390 | 0.063 | 0.760 |
|  |  | rs1260326 | 0.007 | 0.008 | 0.062 | 0.021 | 0.660 | 0.980 | 0.003 |
|  |  | rs13233571 | 0.560 | 0.550 | 0.350 | 0.730 | 0.840 | 0.390 | 0.280 |
|  |  | rs1800947 | 0.950 | 0.560 | NA | 0.370 | 0.540 | 0.520 | 0.140 |
|  |  | rs2794520 | 0.590 | 0.700 | 0.610 | 0.040 | 0.650 | 0.015 | 0.500 |
|  |  | rs2847281 | 0.820 | 0.670 | 0.810 | 0.058 | 0.360 | 0.790 | 0.750 |
|  |  | rs3093077 | 0.190 | 0.590 | NA | 0.190 | 0.950 | 0.058 | 0.550 |
|  |  | rs340029 | 0.220 | 0.720 | 0.092 | 0.190 | 0.560 | 0.610 | 0.450 |
|  |  | rs4129267 | 0.100 | 0.420 | 0.290 | 0.120 | 0.160 | 0.080 | 0.230 |
|  |  | rs4420065 | 0.710 | 0.780 | 0.480 | 0.110 | 0.400 | 0.096 | 0.980 |
|  |  | rs4420638 | 0.480 | 0.000 | 0.048 | 0.680 | 0.110 | 0.770 | 0.000 |
|  |  | rs4705952 | 0.810 | 0.140 | 0.970 | 0.410 | 0.690 | 0.047 | 0.610 |
|  |  | rs6734238 | 0.760 | 0.810 | 0.540 | 0.290 | 0.300 | 0.860 | 0.580 |
|  |  | rs6901250 | 0.034 | 0.400 | 0.270 | 0.750 | 0.990 | 0.069 | 0.510 |
|  |  | rs9987289 | 0.008 | 0.620 | NA | 0.006 | 0.170 | 0.610 | 0.810 |
| IL-6 | Naitza *et al.* | rs579459 | 0.015 | 0.001 | 0.450 | 0.630 | 0.900 | 0.500 | 0.007 |
|  |  | rs630014 | 0.004 | 0.370 | 0.023 | 0.270 | 0.850 | 0.220 | 0.440 |
|  |  | rs643434 | 0.003 | 0.410 | 0.710 | 0.100 | 0.720 | 0.360 | 0.170 |
|  |  | rs651007 | 0.013 | 0.003 | 0.370 | 0.470 | 0.610 | 0.440 | 0.011 |
|  |  | rs687289 | 0.003 | 0.550 | 0.930 | 0.039 | 0.790 | 0.570 | 0.180 |
|  | IL6RMR | rs4845371 | 0.081 | 0.940 | 0.160 | 0.680 | 0.150 | 0.061 | 0.860 |
|  |  | rs7529229 | 0.350 | 0.180 | NA | 0.260 | 0.190 | 0.084 | 0.380 |
| ESR | Naitza *et al.* | rs11117956 | 0.380 | 0.640 | 0.140 | 0.790 | 0.970 | 0.240 | 0.370 |
|  |  | rs12034598 | 0.810 | 0.900 | 0.620 | 0.620 | 0.500 | 0.750 | 0.570 |
|  |  | rs3886100 | 0.710 | 0.380 | 0.990 | 0.670 | 0.240 | 0.610 | 0.680 |
|  |  | rs7519119 | 0.980 | 0.540 | 0.550 | 0.420 | 0.370 | 0.820 | 0.430 |
| MCP-1 | Naitza *et al.* | rs10489849 | 0.520 | 0.130 | 0.044 | 0.640 | 0.660 | 0.430 | 0.210 |
|  |  | rs11265142 | 0.950 | 0.390 | 0.280 | 0.650 | 0.410 | 0.550 | 0.690 |
|  |  | rs11265177 | 0.071 | 0.570 | 0.480 | 0.980 | 0.950 | 0.007 | 0.340 |
|  |  | rs11265186 | 0.650 | 0.470 | 0.860 | 0.810 | 0.710 | 0.230 | 0.200 |
|  |  | rs12047230 | 0.340 | 0.120 | 0.320 | 0.460 | 0.850 | 0.093 | 0.370 |
|  |  | rs12075 | 0.031 | 0.620 | 0.077 | 0.210 | 0.970 | 0.280 | 0.880 |
|  |  | rs12087465 | 0.380 | 0.870 | 0.037 | 0.130 | 0.400 | 0.290 | 0.820 |
|  |  | rs1446954 | 0.720 | 0.770 | 0.031 | 0.066 | 0.150 | 0.520 | 0.810 |
|  |  | rs1474747 | 0.600 | 0.950 | 0.180 | 1.000 | 0.790 | 0.650 | 0.950 |
|  |  | rs1584252 | 0.450 | 0.550 | 0.530 | 0.350 | 0.790 | 0.290 | 0.800 |
|  |  | rs16841987 | 0.170 | 0.008 | 0.016 | 0.170 | 0.940 | 0.260 | 0.020 |
|  |  | rs17666424 | 0.200 | 0.065 | 0.007 | 0.320 | 0.920 | 0.280 | 0.074 |
|  |  | rs2427825 | 0.990 | 0.980 | 0.830 | 0.610 | 0.550 | 0.320 | 0.580 |
|  |  | rs2494261 | 0.960 | 0.910 | 0.410 | 0.510 | 0.350 | 0.380 | 0.340 |
|  |  | rs2592881 | 0.940 | 0.830 | 0.069 | 0.040 | 0.150 | 0.400 | 0.930 |
|  |  | rs3026946 | 0.620 | 0.800 | 0.970 | 0.800 | 0.500 | 0.500 | 0.890 |
|  |  | rs3026968 | 0.940 | 0.340 | 0.550 | 0.440 | 0.360 | 0.810 | 0.430 |
|  |  | rs3027012 | 0.420 | 0.820 | 0.500 | 0.550 | 0.720 | 0.990 | 0.370 |
|  |  | rs3027031 | 0.410 | 0.120 | 0.280 | 0.990 | 0.830 | 0.160 | 0.058 |
|  |  | rs3806185 | 0.066 | 0.990 | 0.120 | 0.200 | 0.490 | 0.520 | 0.780 |
|  |  | rs4656237 | 0.700 | 0.440 | 0.210 | 0.870 | 0.660 | 0.240 | 0.170 |
|  |  | rs6660102 | 0.460 | 0.100 | 0.031 | 0.310 | 0.750 | 0.460 | 0.099 |

**BMI**: body mass index;

**WHRadjBMI**: waist-to-hip-ratio adjusted for BMI;

**Extreme** **WHRadjBMI**: upper 5th percentile (case) vs. lower 5th percentile (control);

**HIPadjBMI**: hip circumference adjusted for BMI;

**WCadjBMI**: waist circumference adjusted for BMI.

**Table 2** Mendelian randomization (MR) analysis of the effects on obesity-related traits of the single nucleotide polymorphisms (SNPs) predicting inflammation

| **Inflammatory marker** | **Source** | **SNP** | **Obesity-related trait** | **Sex** | **β** | **P-value^a^** |
| --- | --- | --- | --- | --- | --- | --- |
| hsCRP | Prins *et al.* | rs1260326 | BMI | Men | -0.228 | 1.6E-4 |
|  |  |  | WCadjBMI | Women | 0.194 | 0.004 |
|  |  |  | Weight | Men | -0.361 | 1.2E-4 |
|  |  |  | WHRadjBMI | Men | 0.264 | 0.007 |
|  |  |  | WHRadjBMI | Women | 0.250 | 0.009 |
|  |  | rs13233571 | Obesity, class 2 | Both | 1.741 | 0.010 |
|  |  | rs2847281 | Obesity, overweight | Both | -0.903 | 0.008 |
|  |  | rs4129267 | BMI | Men | -0.168 | 0.001 |
| IL-6 | Naitza *et al.* | rs630014 | Weight | Men | 0.133 | 0.008 |
|  |  |  | WHRadjBMI | Men | -0.140 | 0.009 |
|  |  | rs651007 | WHRadjBMI | Women | 0.118 | 0.006 |
|  |  | rs687289 | WHRadjBMI | Men | -0.087 | 0.005 |

**BMI**: body mass index;

**WCadjBMI**: waist circumference adjusted for BMI;

**WHRadjBMI**: waist-to-hip-ratio adjusted for BMI;

**Obesity, class 2**: BMI ≥35 (case) vs. BMI <25 (control);

**Obesity, overweight**: BMI ≥25 (case) vs. BMI <25 (control);

^a^ A p-value threshold of 0.0125 was used because the MR tested four inflammatory markers, i.e. hsCRP, IL-6, ESR, and MCP-1.

**Table 3** Mendelian randomization (MR) analysis (IVW) of inflammatory markers and obesity-related traits

| **Inflammatory marker** | **Source** | **Obesity-related trait** | **Sex** | **β** | **P-value^a^** |
| --- | --- | --- | --- | --- | --- |
| IL-6 | Naitza et al. | WHRadjBMI | Men | -0.080 | 0.006 |
|  | IL6RMR | WCadjBMI | Men | -0.189 | 2.5E-6 |
|  |  | WHRadjBMI | Men | -0.205 | 3.6E-4 |
| ESR | Naitza et al. | Obesity, class 1 | Both | 0.174 | 0.006 |
|  |  | Obesity, overweight | Both | 0.113 | 0.011 |
| MCP-1 | Naitza et al. | BMI | Men | 0.020 | 7.2E-7 |
|  |  | Childhood obesity | Both | -0.132 | 2.0E-7 |
|  |  | Extreme WHRadjBMI | Both | -0.102 | 0.006 |
|  |  | HIPadjBMI | Men | -0.043 | 5.1E-6 |
|  |  | Obesity, class 1 | Both | -0.032 | 1.4E-4 |
|  |  | Obesity, class 3 | Both | 0.160 | 6.4E-10 |
|  |  | Obesity, overweight | Both | -0.021 | 0.001 |
|  |  | WCadjBMI | Women | -0.021 | 1.9E-5 |
|  |  | Weight | Men | 0.032 | 1.5E-8 |

**BMI**: body mass index;

**WHRadjBMI**: waist-to-hip-ratio adjusted for BMI;

**WCadjBMI**: waist circumference adjusted for BMI;

**Obesity, class1**: BMI ≥30 (case) vs. BMI <25 (control);

**Obesity, overweight**: BMI ≥25 (case) vs. BMI <25 (control);

**Childhood obesity**: ≥95th percentile of BMI (case) and <50th percentile of BMI (control);

**Extreme WHRadjBMI**: upper 5th percentile (case) vs. lower 5th percentile (control) (adjusted for BMI);

**HIPadjBMI**: hip circumference adjusted for BMI;

**Obesity, class1**: BMI ≥30 (case) vs. BMI <25 (control);

**Obesity class 3**: BMI ≥40 (case) vs. BMI <25 (control);

^a^ A p-value threshold of 0.0125 was used because the MR tested two inflammatory markers, i.e. hsCRP, IL-6, ESR, and MCP-1.


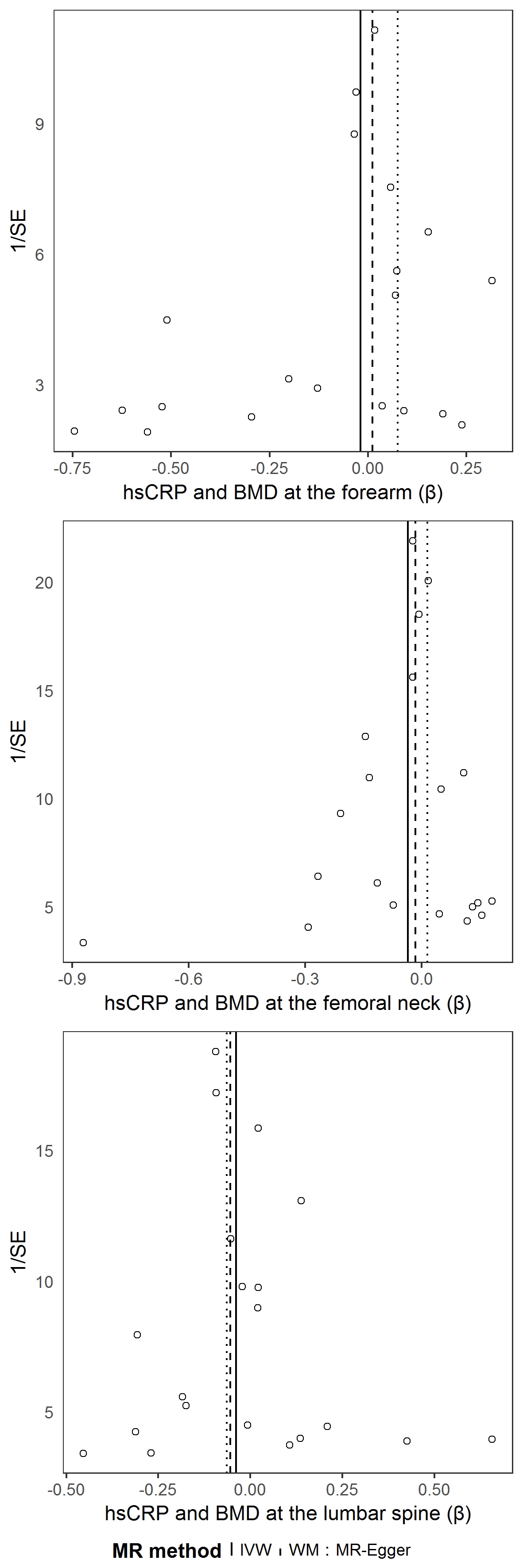


Figure 1 Funnel plots of genetic association with hsCRP (Prins *et al.*) against SNP-specific causal effect on bone mineral density (BMD)


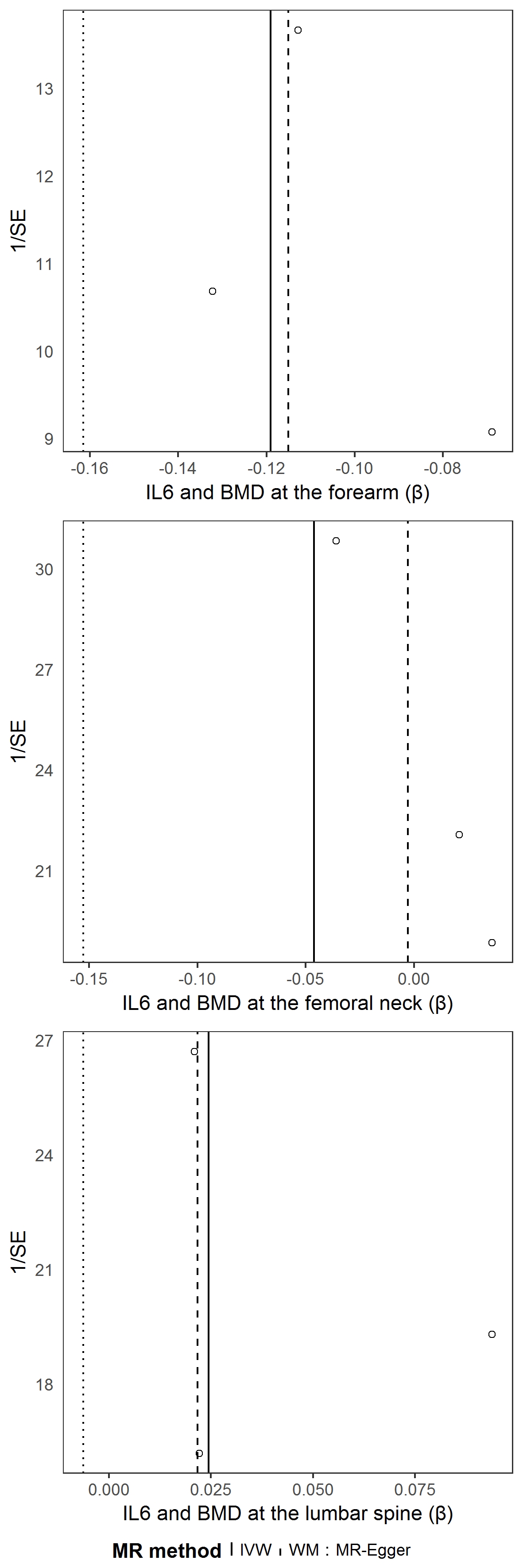

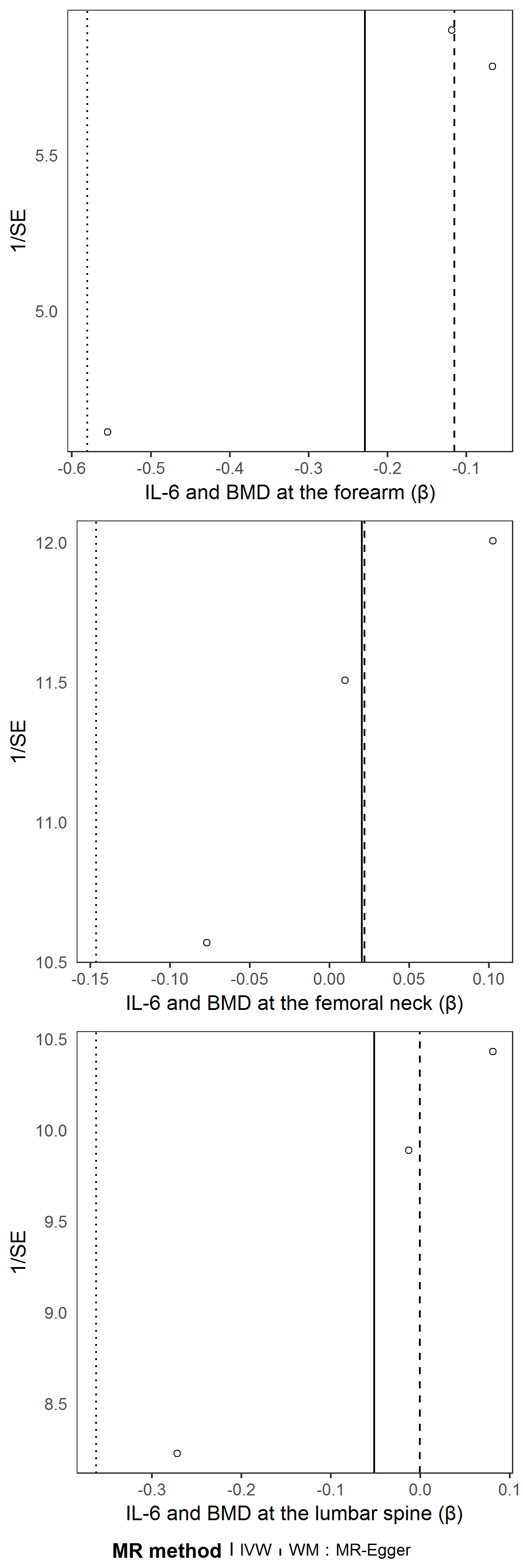

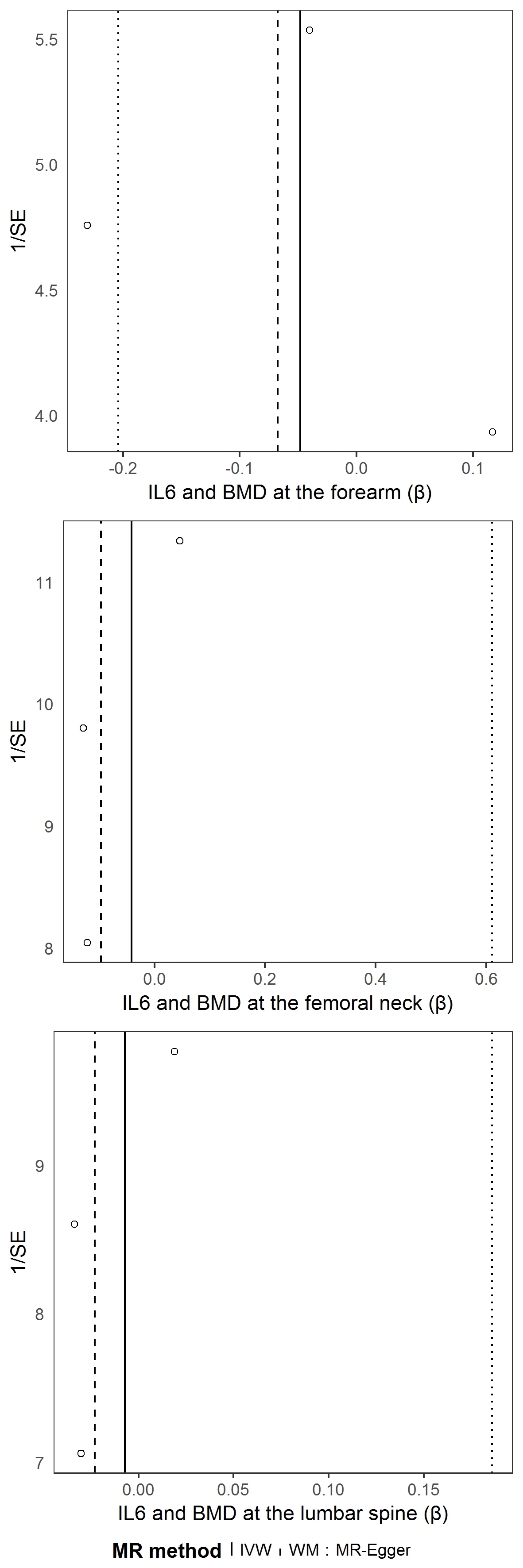


Figure 2 Funnel plots of genetic association with IL-6 (Left: Naitza *et al.*; Middle: Ahola-Olli *et al*.; Right: IL-6R MR Analysis Consortium) against SNP-specific causal effect on bone mineral density (BMD)


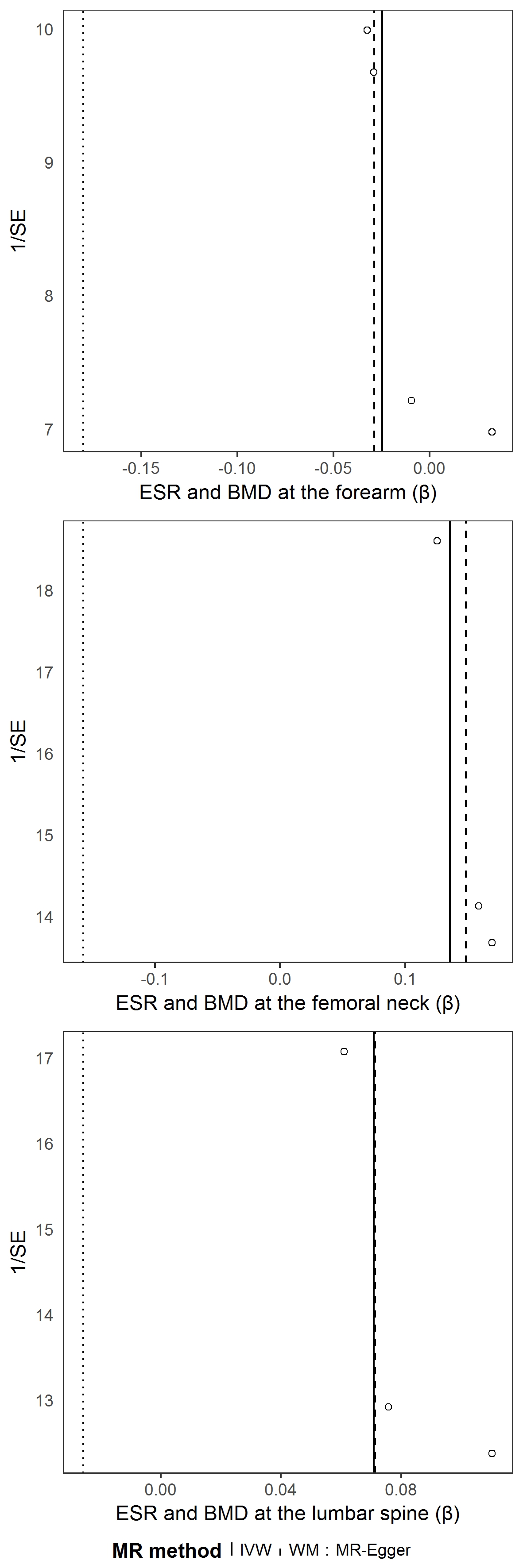


**Figure 3** Funnel plots of genetic association with ESR (Naitza *et al.*) against SNP-specific causal effect on bone mineral density (BMD)


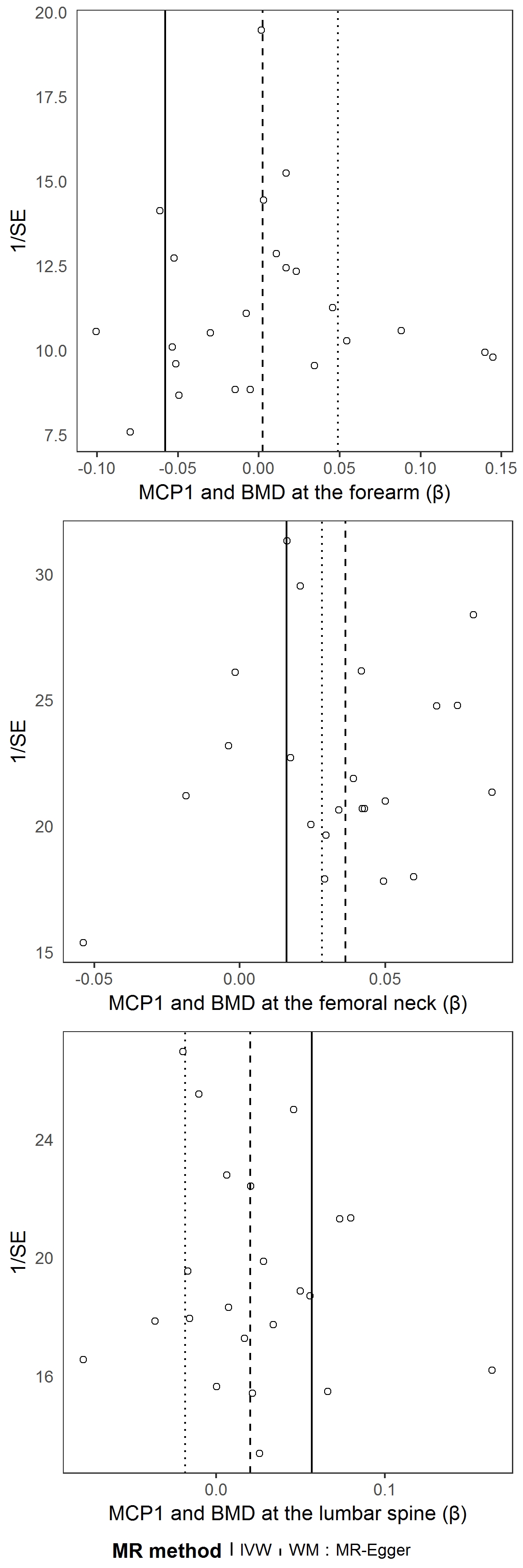


Figure 4 Funnel plots of genetic association with MCP-1 (Naitza *et al.*) against SNP-specific causal effect on bone mineral density (BMD)


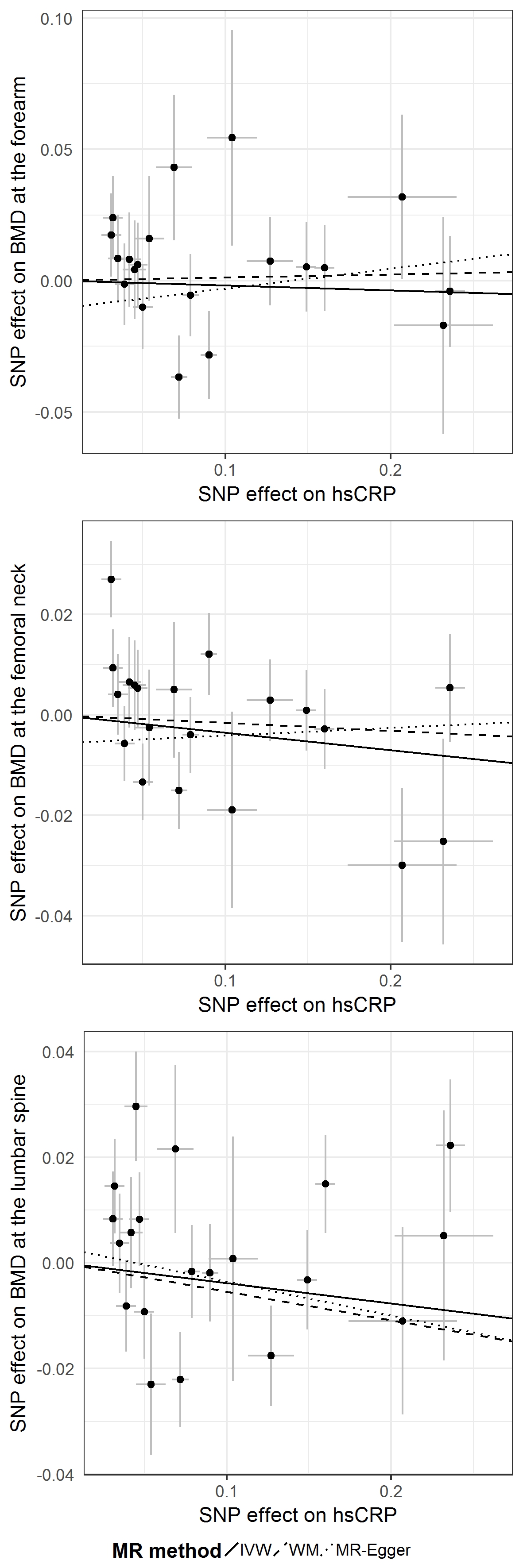


Figure 5 Scatter plots of the association of SNP effect on hsCRP (Prins *et al.*) with SNP effect on bone mineral density (BMD)


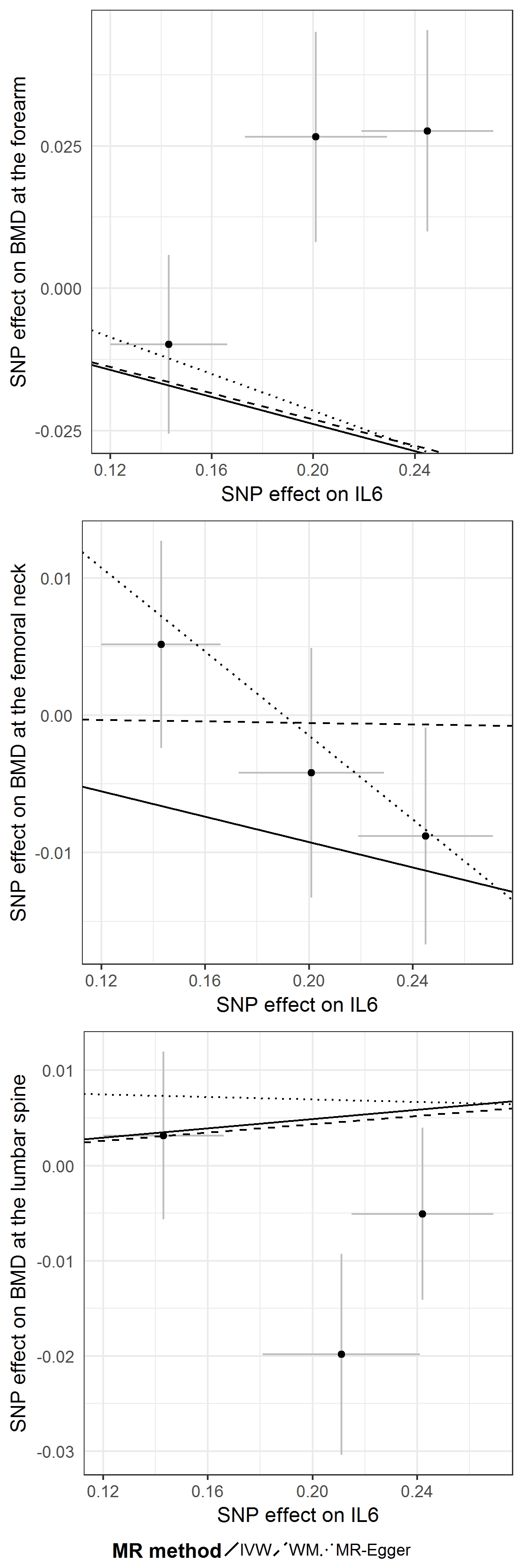

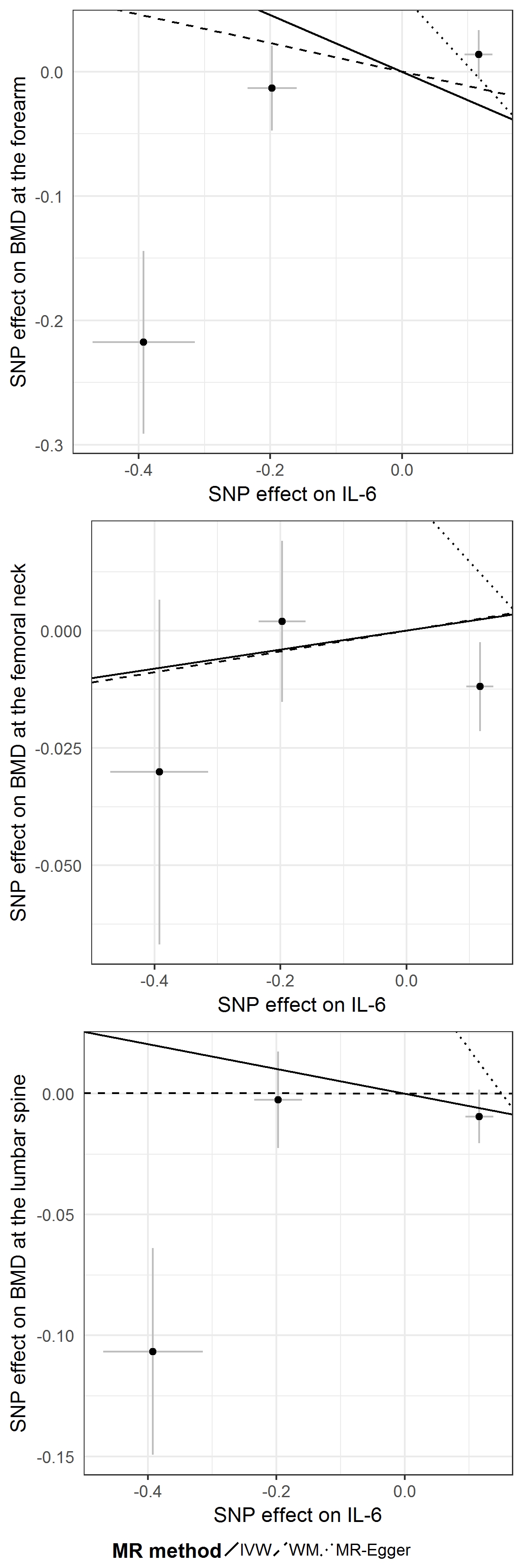

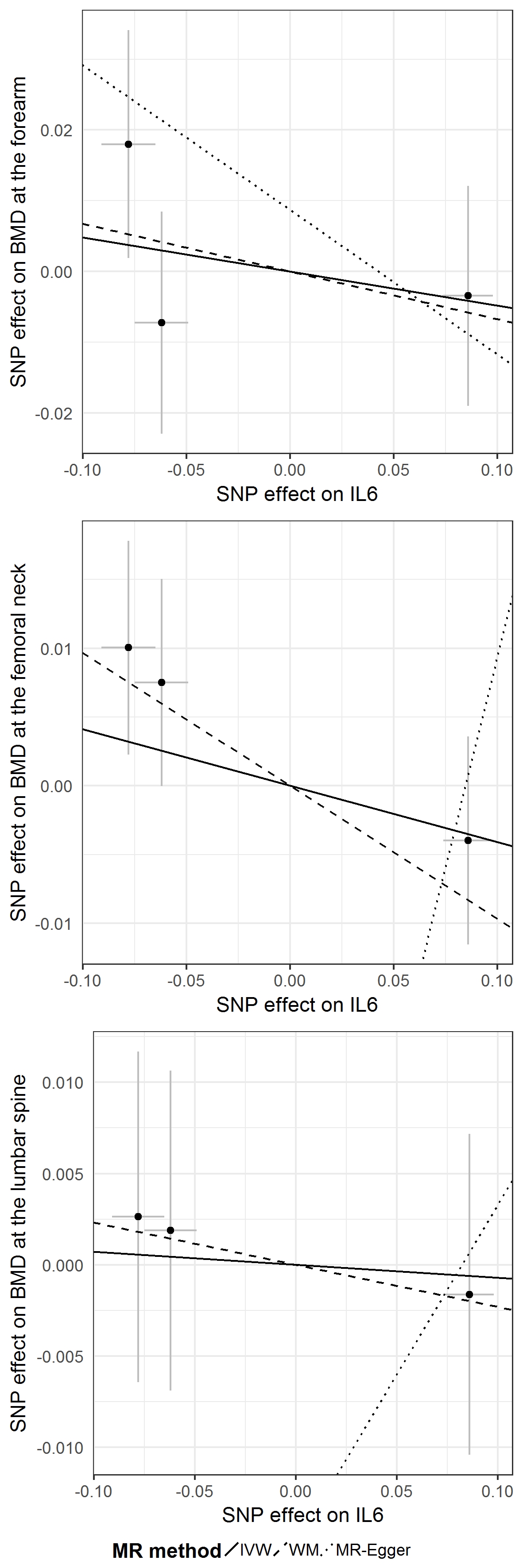


Figure 6 Scatter plots of the association of SNP effect on IL-6 (Left: Naitza *et al.*; Middle: Ahola-Olli *et al*.; Right: IL-6R MR Analysis Consortium) with SNP effect on bone mineral density (BMD)


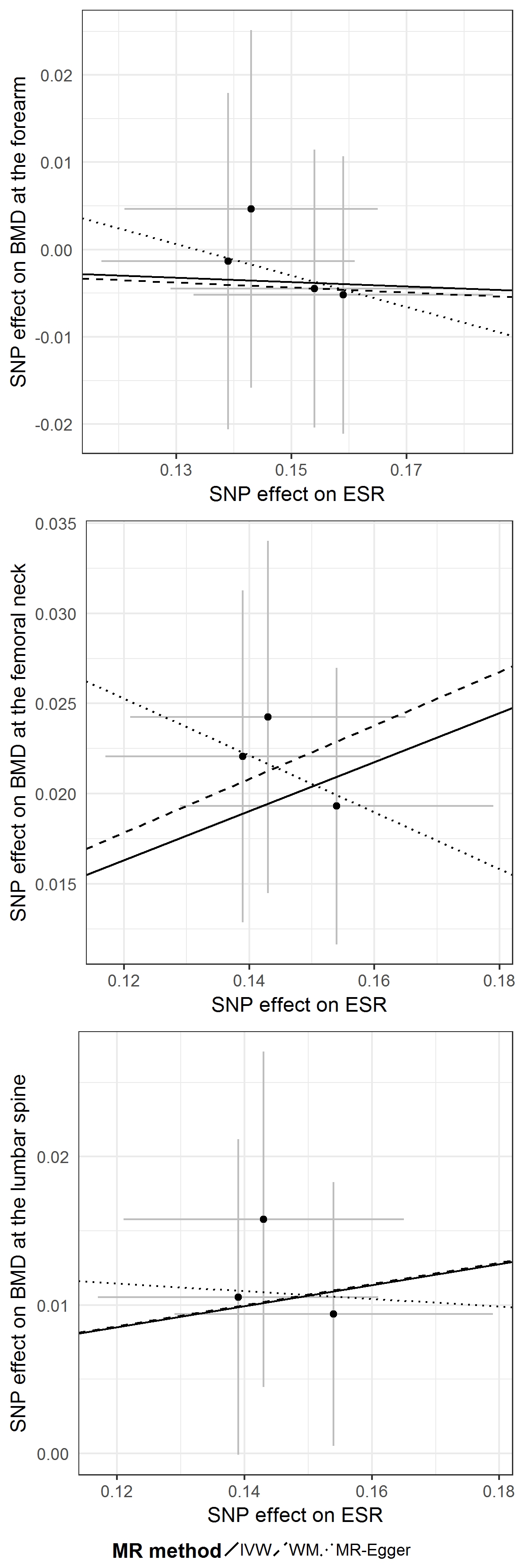


Figure 7 Scatter plots of the association of SNP effect on ESR (Naitza *et al.*) with SNP effect on bone mineral density (BMD)


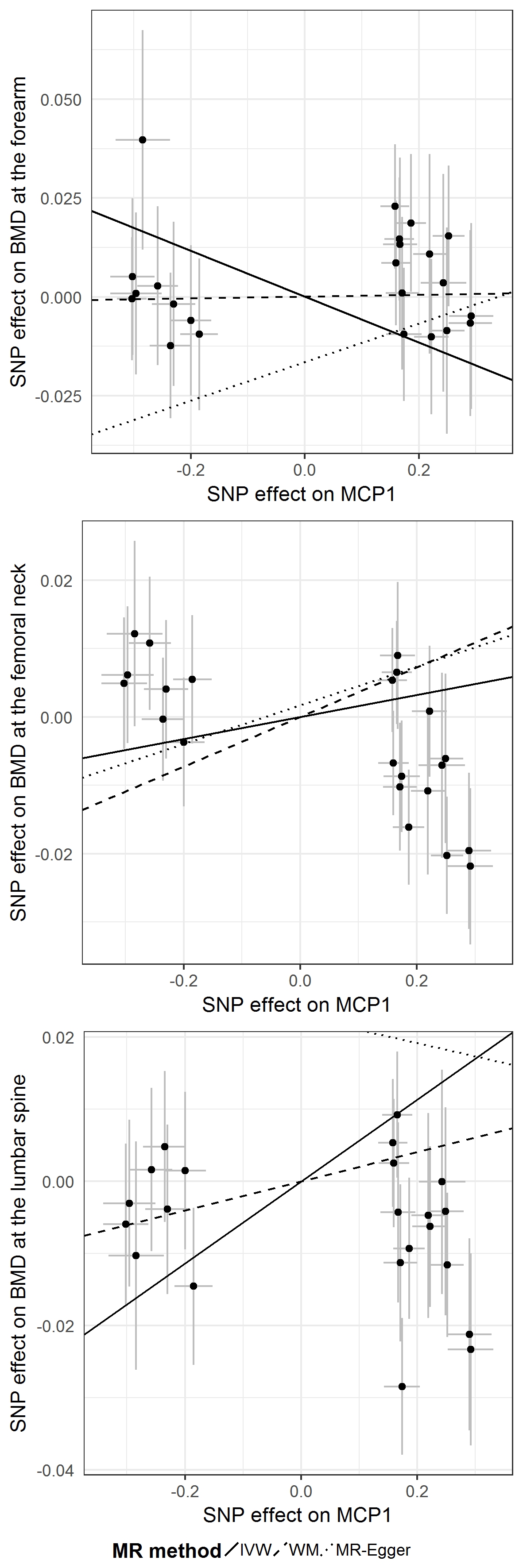


Figure 8 Scatter plots of the association of SNP effect on MCP-1 (Naitza *et al.*) with SNP effect on bone mineral density (BMD)

Table 4 Estimates of the effects of inflammation markers (IL-6, ESR, and, MCP-1) on bone mineral density (in standard deviations) at forearm, femoral neck, and lumbar spine provided by GEFOS

| **Inflammatory marker** | **GWAS^a^** | **Skeletal site (GEFOS 2015)^b^** | **All SNPs** | | | | | |  | **Excluding potentially pleiotropic SNPs^f^** | | | | | |
| --- | --- | --- | --- | --- | --- | --- | --- | --- | --- | --- | --- | --- | --- | --- | --- |
|  |  |  | **No. of SNPs^c^** | **Method^d^** | **β** | **P-value^e^** | **MR-Egger** | |  | **No. of SNPs^b^** | **Method** | **β** | **P-value^e^** | **MR-Egger** | |
|  |  |  |  |  |  |  | **Intercept** | **P-value** |  |  |  |  |  | **Intercept** | **P-value** |
| IL-6 | Naitza *et al.* | Forearm | 3 | IVW | -0.119 | 0.096 |  |  |  | 2 | IVW | -0.115 | 0.109 |  |  |
|  |  |  | 3 | WM | -0.115 | 0.042 |  |  |  |  |  |  |  |  |  |
|  |  |  | 3 | MR-Egger | -0.162 | 0.177 | 0.011 | 0.658 |  |  |  |  |  |  |  |
|  |  | Femoral neck | 3 | IVW | -0.046 | 0.147 |  |  |  | 2 | IVW | -0.036 | 0.266 |  |  |
|  |  |  | 3 | WM | -0.003 | 0.916 |  |  |  |  |  |  |  |  |  |
|  |  |  | 3 | MR-Egger | **-0.153** | **0.005** | 0.029 | 0.016 |  |  |  |  |  |  |  |
|  |  | Lumbar spine | 3 | IVW | 0.024 | 0.507 |  |  |  |  |  |  |  |  |  |
|  |  |  | 3 | WM | 0.022 | 0.487 |  |  |  |  |  |  |  |  |  |
|  |  |  | 3 | MR-Egger | -0.006 | 0.946 | 0.008 | 0.690 |  |  |  |  |  |  |  |
|  | Ahola-Olli *et al*. | Forearm | 3 | IVW | **-0.229** | **0.024** |  |  |  |  |  |  |  |  |  |
|  |  |  | 3 | WM | -0.115 | 0.409 |  |  |  |  |  |  |  |  |  |
|  |  |  | 3 | MR-Egger | -0.580 | 0.053 | 0.063 | 0.200 |  |  |  |  |  |  |  |
|  |  | Femoral neck | 3 | IVW | 0.020 | 0.685 |  |  |  |  |  |  |  |  |  |
|  |  |  | 3 | WM | 0.022 | 0.727 |  |  |  |  |  |  |  |  |  |
|  |  |  | 3 | MR-Egger | -0.146 | 0.242 | 0.029 | 0.146 |  |  |  |  |  |  |  |
|  |  | Lumbar spine | 3 | IVW | -0.051 | 0.379 |  |  |  |  |  |  |  |  |  |
|  |  |  | 3 | WM | 0.000 | 0.995 |  |  |  |  |  |  |  |  |  |
|  |  |  | 3 | MR-Egger | **-0.362** | **0.013** | 0.055 | 0.020 |  |  |  |  |  |  |  |
|  | IL6RMR | Forearm | 3 | IVW | -0.048 | 0.327 |  |  |  |  |  |  |  |  |  |
|  |  |  | 3 | WM | -0.068 | 0.632 |  |  |  |  |  |  |  |  |  |
|  |  |  | 3 | MR-Egger | -0.204 | 0.667 | 0.009 | 0.799 |  |  |  |  |  |  |  |
|  |  | Femoral neck | 3 | IVW | -0.041 | 0.084 |  |  |  |  |  |  |  |  |  |
|  |  |  | 3 | WM | -0.097 | 0.165 |  |  |  |  |  |  |  |  |  |
|  |  |  | 3 | MR-Egger | 0.611 | 0.014 | -0.052 | 0.004 |  |  |  |  |  |  |  |
|  |  | Lumbar spine | 3 | IVW | -0.007 | 0.797 |  |  |  |  |  |  |  |  |  |
|  |  |  | 3 | WM | -0.023 | 0.760 |  |  |  |  |  |  |  |  |  |
|  |  |  | 3 | MR-Egger | 0.186 | 0.461 | -0.015 | 0.395 |  |  |  |  |  |  |  |
| ESR | Naitza *et al.* | Forearm | 4 | IVW | -0.025 | 0.724 |  |  |  |  |  |  |  |  |  |
|  |  |  | 4 | WM | -0.029 | 0.673 |  |  |  |  |  |  |  |  |  |
|  |  |  | 4 | MR-Egger | -0.180 | 0.862 | 0.024 | 0.881 |  |  |  |  |  |  |  |
|  |  | Femoral neck | 3 | IVW | **0.136** | **0.004** |  |  |  |  |  |  |  |  |  |
|  |  |  | 3 | WM | **0.149** | **4.9E-4** |  |  |  |  |  |  |  |  |  |
|  |  |  | 3 | MR-Egger | -0.157 | 0.777 | 0.044 | 0.597 |  |  |  |  |  |  |  |
|  |  | Lumbar spine | 3 | IVW | 0.071 | 0.197 |  |  |  |  |  |  |  |  |  |
|  |  |  | 3 | WM | 0.071 | 0.123 |  |  |  |  |  |  |  |  |  |
|  |  |  | 3 | MR-Egger | -0.026 | 0.968 | 0.015 | 0.88 |  |  |  |  |  |  |  |
| MCP-1 | Naitza *et al.* | Forearm | 23 | IVW | **-0.058** | **1.1E-5** |  |  |  | 22 | IVW | -0.021 | 0.437 |  |  |
|  |  |  | 23 | WM | 0.002 | 0.923 |  |  |  | 22 | WM | 0.006 | 0.810 |  |  |
|  |  |  | 23 | MR-Egger | 0.049 | 0.169 | -0.016 | 0.019 |  | 22 | MR-Egger | 0.089 | 0.088 | -0.025 | 0.021 |
|  |  | Femoral neck | 22 | IVW | 0.016 | 0.222 |  |  |  |  |  |  |  |  |  |
|  |  |  | 22 | WM | **0.036** | **0.003** |  |  |  |  |  |  |  |  |  |
|  |  |  | 22 | MR-Egger | 0.028 | 0.265 | 0.002 | 0.746 |  |  |  |  |  |  |  |
|  |  | Lumbar spine | 22 | IVW | **0.057** | **0.002** |  |  |  |  |  |  |  |  |  |
|  |  |  | 22 | WM | 0.020 | 0.177 |  |  |  |  |  |  |  |  |  |
|  |  |  | 22 | MR-Egger | -0.018 | 0.621 | 0.023 | 0.003 |  |  |  |  |  |  |  |

^a^ In the GWAS of Naitza *et al*., inflammatory markers were analysed in a SD unit, SD of IL-6 was 2.8 pg/mL, SD of ESR was 7.2 mm/h, and SD of MCP-1 was 121.4 pg/mL; in the MR study of IL-6R MR Analysis Consortium, IL-6 was analysed on a natural log scale in pg/mL;

^b^ Bone mineral density was measured as the standard deviation (SD) from the healthy young adult reference;

^c^ Number of SNPs included in the analysis of one markers at different skeletal sites may differ, because particular SNPs may not be available in GEFOS of specific skeletal site;

^d^ Inverse-variance weighted (IVW), weighted median (WM), and MR-Egger were performed for testing the robustness of the association; WM and MR-Egger are only feasible with more than two SNPs;

^e^ Associations with a p-value smaller than 0.0125 were in bold;

^f^ SNPs with potentially pleiotropic effects related to obesity were excluded from the sensitivity analysis, including rs630014 (*ABO*), rs651007 (*ABO*), and rs687289 (*ABO*) for IL-6; and rs12075 (*DARC*/*CADM3*) for MCP-1; sensitivity analysis was not performed at the skeletal sites that the SNP with potential pleiotropic effect was already excluded in corresponding “All SNPs” analysis; sensitivity analysis was not performed for the association of IL-6 with BMD at the lumbar spine since all three SNPs from the GWAS of Naitza *et al*. have potentially pleiotropic effects.


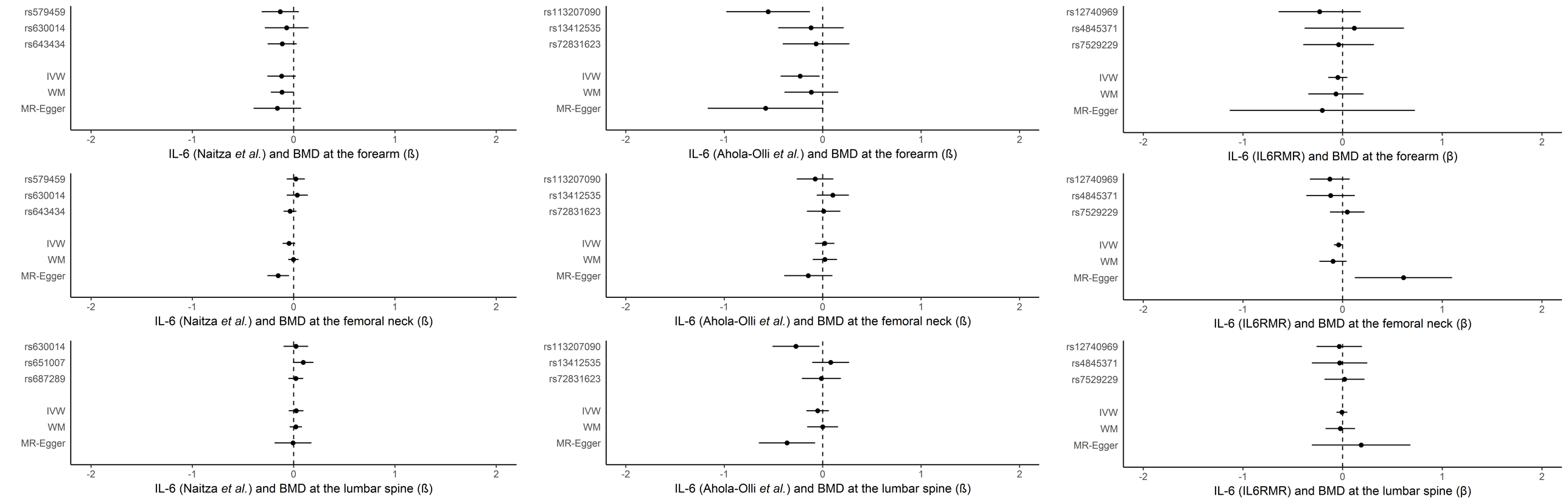


Figure 9 SNP-specific associations of IL-6 with BMD at each skeletal site (Left: Naitza *et al*.; Middle: Ahola-Olli *et al*.; Right: IL-6R MR Analysis Consortium)


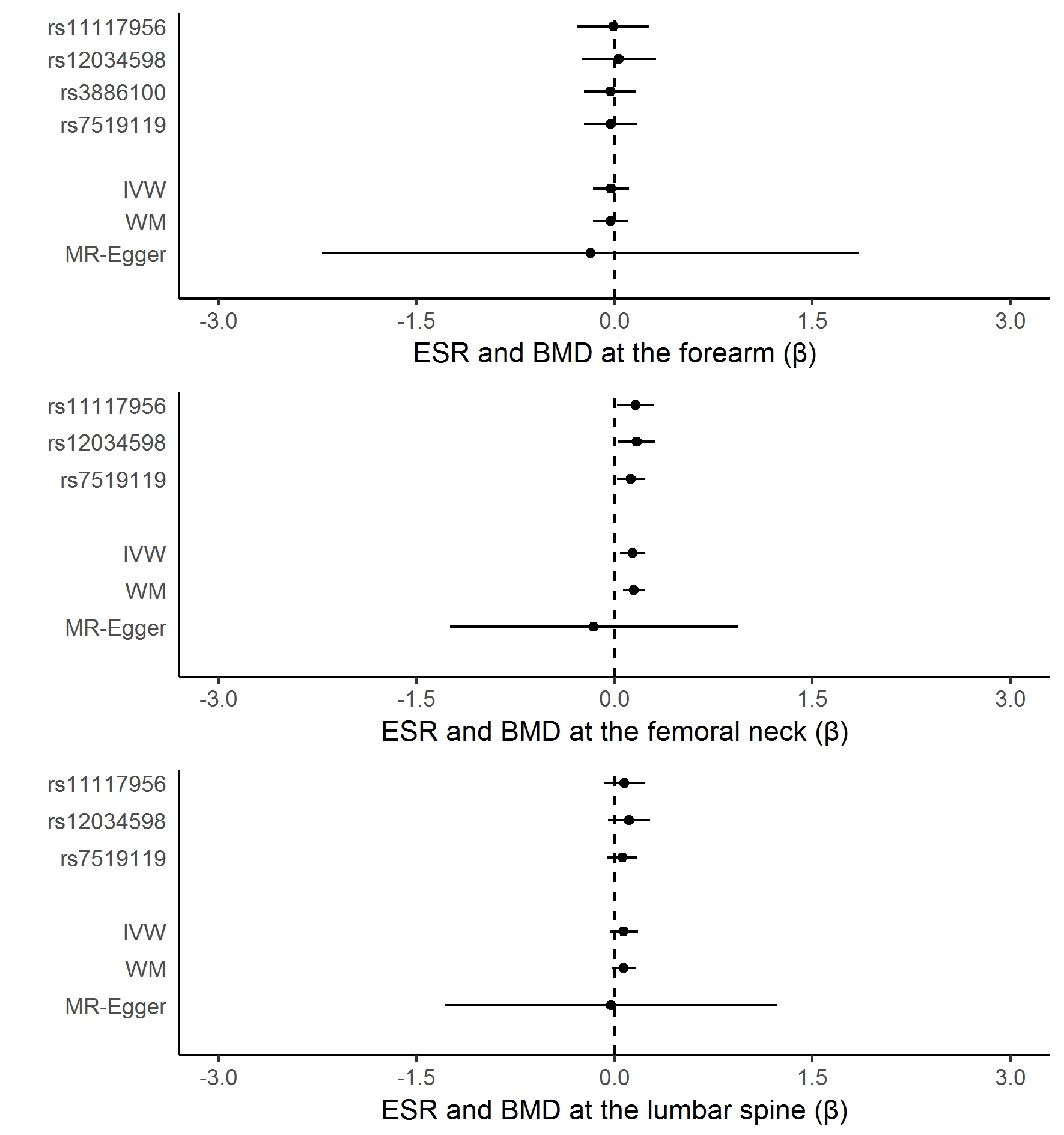


Figure 10 SNP-specific associations of ESR (Naitza *et al*.) with BMD at each skeletal site


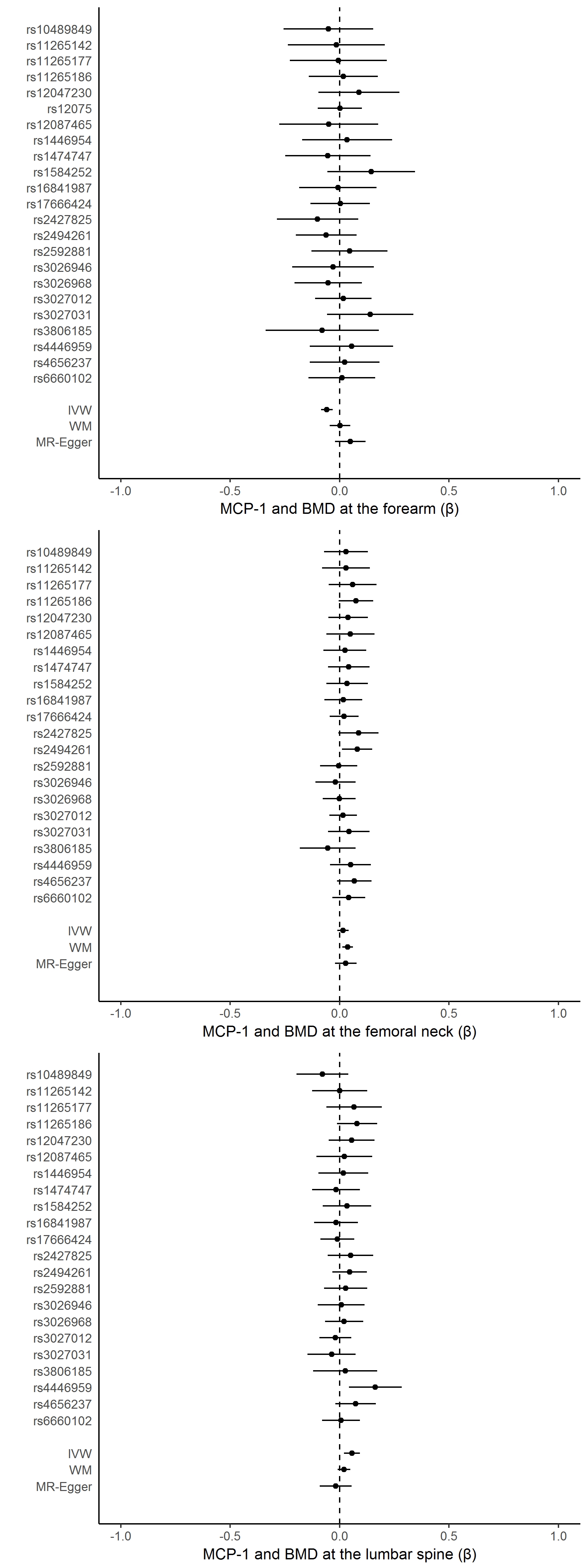


Figure 11 SNP-specific associations of MCP-1 (Naitza *et al*.) with bone mineral density (BMD) at each skeletal site

**Table 5 Estimation of R^2^ and F-statistics of SNPs predicting inflammatory markers**

| **Phenotype** | **Source** | **SNP** | **R^2^** | **F-statistic** |
| --- | --- | --- | --- | --- |
| hsCRP | Prins *et al.* | rs10521222 | 0.002 | 431 |
|  |  | rs10745954 | 0.001 | 198 |
|  |  | rs1130864 | 0.007 | 1808 |
|  |  | rs1183910 | 0.009 | 2413 |
|  |  | rs12037222 | 0.001 | 191 |
|  |  | rs12239046 | 0.001 | 262 |
|  |  | rs1260326 | 0.002 | 650 |
|  |  | rs13233571 | 0.001 | 157 |
|  |  | rs1800947 | 0.007 | 1758 |
|  |  | rs2794520 | 0.011 | 2999 |
|  |  | rs2847281 | 0.0004 | 117 |
|  |  | rs3093077 | 0.004 | 1065 |
|  |  | rs340029 | 0.0005 | 123 |
|  |  | rs4129267 | 0.003 | 734 |
|  |  | rs4420065 | 0.004 | 964 |
|  |  | rs4420638 | 0.017 | 4419 |
|  |  | rs4705952 | 0.0005 | 126 |
|  |  | rs6734238 | 0.001 | 317 |
|  |  | rs6901250 | 0.001 | 141 |
|  |  | rs9987289 | 0.001 | 172 |
| IL-6 | Naitza *et al.* | rs579459 | 0.012 | 50 |
|  |  | rs630014 | 0.010 | 42 |
|  |  | rs643434 | 0.023 | 101 |
|  |  | rs651007 | 0.013 | 55 |
|  |  | rs687289 | 0.021 | 91 |
|  | Ahola-Olli *et al*.^a^ | rs113207090 | 0.002 | 3 |
|  |  | rs13412535 | 0.005 | 8 |
|  |  | rs72831623 | 0.005 | 8 |
|  | IL6RMR | rs12740969 | 0.003 | 795 |
|  |  | rs4845371 | 0.002 | 492 |
|  |  | rs7529229 | 0.004 | 958 |
| ESR | Naitza *et al.* | rs11117956 | 0.009 | 34 |
|  |  | rs12034598 | 0.010 | 36 |
|  |  | rs3886100 | 0.009 | 32 |
|  |  | rs7519119 | 0.009 | 32 |
| MCP-1 | Naitza *et al.* | rs10489849 | 0.009 | 41 |
|  |  | rs11265142 | 0.011 | 50 |
|  |  | rs11265177 | 0.011 | 49 |
|  |  | rs11265186 | 0.017 | 75 |
|  |  | rs12047230 | 0.013 | 57 |
|  |  | rs12075 | 0.046 | 206 |
|  |  | rs12087465 | 0.016 | 69 |
|  |  | rs1446954 | 0.018 | 80 |
|  |  | rs1474747 | 0.012 | 53 |
|  |  | rs1584252 | 0.012 | 52 |
|  |  | rs16841987 | 0.009 | 40 |
|  |  | rs17666424 | 0.014 | 61 |
|  |  | rs2427825 | 0.015 | 66 |
|  |  | rs2494261 | 0.025 | 108 |
|  |  | rs2592881 | 0.016 | 71 |
|  |  | rs3026946 | 0.010 | 41 |
|  |  | rs3026968 | 0.013 | 55 |
|  |  | rs3027012 | 0.018 | 81 |
|  |  | rs3027031 | 0.011 | 49 |
|  |  | rs3806185 | 0.009 | 40 |
|  |  | rs4446959 | 0.010 | 41 |
|  |  | rs4656237 | 0.017 | 74 |
|  |  | rs6660102 | 0.016 | 70 |

^a^ Effect allele frequency was not provided in the GWAS dataset of Ahola-Olli *et al*. For the three SNPs from this study, we obtained the effect allele frequency in Ensembl, based on Finnish population in Finland or European population.

**Reference:**

1. Berndt SI, Gustafsson S, Magi R, et al. Genome-wide meta-analysis identifies 11 new loci for anthropometric traits and provides insights into genetic architecture. *Nat Genet* 2013; **45**: 501-12.

2. Randall JC, Winkler TW, Kutalik Z, et al. Sex-stratified genome-wide association studies including 270,000 individuals show sexual dimorphism in genetic loci for anthropometric traits. *PLoS genetics* 2013; **9**: e1003500.

3. Locke AE, Kahali B, Berndt SI, et al. Genetic studies of body mass index yield new insights for obesity biology. *Nature* 2015; **518**: 197-206.

4. Bradfield JP, Taal HR, Timpson NJ, et al. A genome-wide association meta-analysis identifies new childhood obesity loci. *Nat Genet* 2012; **44**: 526-31.
